# Increasing atmospheric CO_2_ concentrations correlate with declining nutritional status of European forests

**DOI:** 10.1038/s42003-020-0839-y

**Published:** 2020-03-13

**Authors:** Josep Penuelas, Marcos Fernández-Martínez, Helena Vallicrosa, Joan Maspons, Paolo Zuccarini, Jofre Carnicer, Tanja G. M. Sanders, Inken Krüger, Michael Obersteiner, Ivan A. Janssens, Philippe Ciais, Jordi Sardans

**Affiliations:** 1 CSIC, Global Ecology Unit CREAF-CSIC-UAB, 08913 Bellaterra, Catalonia Spain; 2 CREAF, 08913 Cerdanyola del Vallès, Catalonia Spain; 3 Research group PLECO (Plants and Ecosystems), Department of Biology, University of Antwerp, 2610 Wilrijk, Belgium; 4 Thünen Institute of Forest Ecosystems, Alfred-Möller-Straße 1, Haus 41/42, Eberswalde, 16225 Germany; 5 International Institute for Applied Systems Analysis (IIASA), Ecosystems Services and Management, Schlossplatz 1, A-2361 Laxenburg, Austria; 6 Laboratoire des Sciences du Climat et de l’Environnement, IPSL, 91191 Gif-sur-Yvette, France

**Keywords:** Forest ecology, Plant sciences

## Abstract

The drivers of global change, including increases in atmospheric CO_2_ concentrations, N and S deposition, and climate change, likely affect the nutritional status of forests. Here we show forest foliar concentrations of N, P, K, S and Mg decreased significantly in Europe by 5%, 11%, 8%, 6% and 7%, respectively during the last three decades. The decrease in nutritional status was especially large in Mediterranean and temperate forests. Increasing atmospheric CO_2_ concentration was well correlated with the decreases in N, P, K, Mg, S concentrations and the increase of N:P ratio. Regional analyses indicated that increases in some foliar nutrient concentrations such as N, S and Ca in northern Europe occurred associated with increasingly favourable conditions of mean annual precipitation and temperature. Crucial changes in forest health, structure, functioning and services, including negative feedbacks on C capture can be expected if these trends are not reversed.

## Abstract (web summary)

Penuelas et al analyse foliar concentrations of nutrients in the main tree species of European forests using data collected over 30 years, together with changes in atmospheric CO_2_ levels, N and S deposition and climate. They find that foliar concentrations of N, P, K, S and Mg have decreased in central and southern Europe, with implications for forest ecosystems.

## Introduction

Atmospheric CO_2_ concentrations and nitrogen (N) and sulfur (S) deposition, together with warming and drought, likely affect the nutritional status of forests^1–6^ and therefore their functioning, structure and ecosystem services^7–9^. Elevated atmospheric CO_2_ concentrations, usually tested at 500–700 ppm, decrease the N and P concentrations of plants^10–13^. Increases in atmospheric CO_2_ concentrations are frequently correlated with higher growth^10^ and more efficient photosynthesis, and thus likely dilute leaf-level nutrient concentrations. Increases in atmospheric CO_2_ concentrations also reduce transpiration^14^ and stomatal conductance^15^, thus also hindering nutrient uptake^16,17^ that may even ultimately limit the initial increase in plant production under the rise of atmospheric CO_2_ concentrations^18–20^. N deposition also increases tree productivity^21,22^ and foliar N concentrations but can decrease foliar P and Mg concentrations^23–25^. Warming tends to increase the mineralisation, cycling and availability of nutrients when water is available^12^, but the consequent increase in growth involves a dilution of nutrients that leads to frequent decreases in foliar N concentrations^26,27^ and increases in C:nutrient ratios^12,28^. Plants at sites not limited by water respond by increasing nutrient uptake^29,30^, but if warming persists or even increases in the long term, nutrients can become limiting^31,32^. Warming in dry environments, though, can increase soil drought, exacerbating limitations of water and nutrients^33,34^. Plants under these conditions respond by activating mechanisms for conserving and taking up water and nutrients but C:nutrient ratios still frequently increase in photosynthetic tissues^7,35–38^.

The concentrations of atmospheric CO_2_ have increased from ~350–360 ppm in the 1990s to the current 410 ppm (in 2019)^39^. The deposition of oxidised N in some regions of the world such as Europe peaked during the 1980s, up to 6–8-fold higher than in 1900, but has since decreased to half its highest value^40,41^. The annual deposition of reduced Nin Europe is currently more than two-fold higher than in 1900^42^. S deposition in Europe has decreased to ~70% of the level in 1900^42^. Europe has also warmed faster than the global average of 0.27 °C per decade during the last three decades, and this warming has varied throughout Europe. Temperatures have risen by 0.48, 0.44 and 0.34 °C per decade in northern, central and southern Europe, respectively, in the period 1979–2010^43,44^.

These increasing CO_2_ concentrations, changes in N and S deposition and climate change have been accompanied by a general decrease in foliar P concentrations and a consequent increase in N:P ratios in recent decades in *Fagus sylvatica*^24,45–47^, *Picea abies* and *Pinus sylvestris*^24,48^ and *Quercus petraea*^24^. Clear general patterns for foliar N concentrations, however, have not been found, with decreases, increases or no changes, depending on species and foliar cohorts^24,49–52^.Likely local, regional or latitudinal differences have not been considered, so these changes in foliar nutrient concentrations have not been consistently attributed to a particular environmental driver or combination of drivers. Furthermore, most reported nutritional changes in plants refer to N and P concentrations, but other important nutrients are key to the nutritional status of trees, such as K, S, Mg and Ca^4^.

We analysed (i) the changes in foliar elemental composition and stoichiometry during the last three decades for the main tree species in forests throughout Europe (Supplementary Fig. 1) at three different spatial scales: over the entire Europe, at different latitudes, and locally, as well as (ii) the empirical relationships of these changes with their possible drivers, i.e. increased atmospheric CO_2_ concentrations, N and S deposition and climate change, using statistical attribution analyses, data available from field experiments and models of the responses of nutrients to these drivers of global change. The results showed that forest foliar concentrations of N, P, K, S and Mg decreased significantly in Europe by 5%, 11%, 8%, 6% and 7%, respectively, and that these decreases were especially large in Mediterranean and temperate forests and mainly related to the rising of atmospheric CO_2_ concentration. In contrast, foliar N, S and Ca concentrations increased associated with increasingly favourable conditions of mean annual precipitation and temperature in boreal forests. Crucial changes in forest health, structure, functioning and services, including negative feedbacks on C capture can be expected if these trends in central and southern European forests are not reversed.

## Results

### Declining nutritional status

**Fig. 1:**
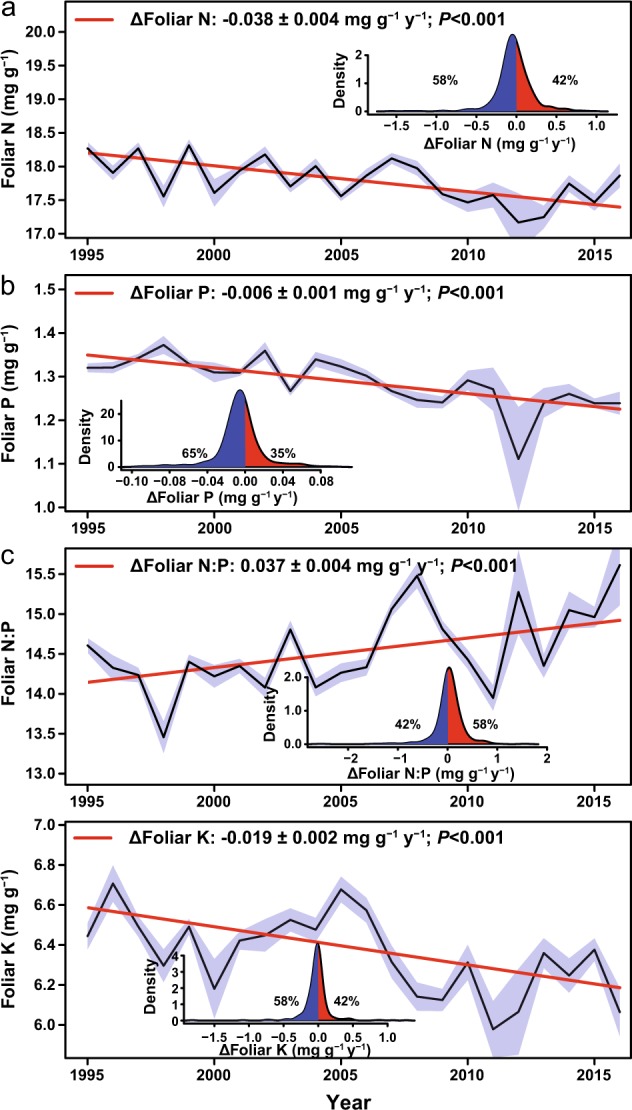
Decreasing tree foliar N, P and K concentrations and increasing N:P ratio. The black lines indicate average trends, and the shaded areas indicate the standard errors of the average trends. The inset shows the percentages of forests with decreasing and increasing foliar nutrient concentrations. Red and blue indicate forests with increasing and decreasing trends, respectively. All values were adjusted to the same mean to remove forest-specific variability. See Supplementary Table 1 for detailed results of the model lme (foliar variable ∼year, random = ∼1|country/plot/species, data=dades, method = “REML”). Statistically significant trends (in percentage of sites): N+: 5.68%, N−: 13.18%; P+: 1.21%, P−: 17.30%; N:P+: 14.72%, N:P−: 4.29%; K+: 2.02%, K−: 9.90%.

**Fig. 2:**
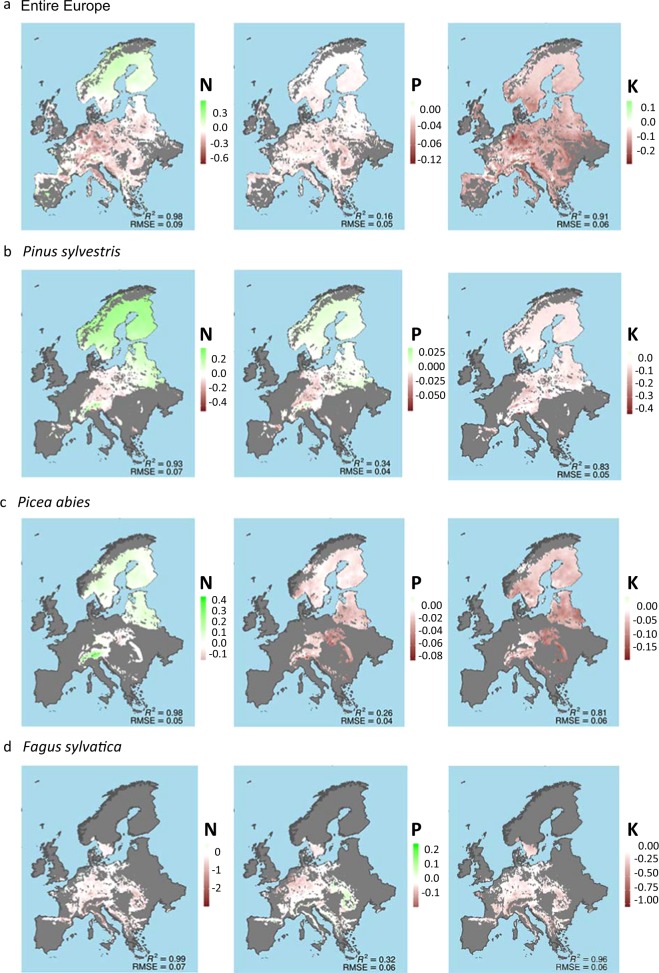
Geographical distribution of the annual rate of change for N, P, and K foliar concentration in mg g^−1^. **a** the entire forests, **b** *Pinus sylvestris*, **c** *Picea abies*, and **d** *Fagus sylvatica*. The estimations are based on neural networks with MAP, MAT and nutrient deposition as inputs, using 80% of the trees with more than five measurements for training and 20% for validation. We replicated the process 1000 times and averaged the results. Map spatial resolution 0.1°.

**Fig. 3:**
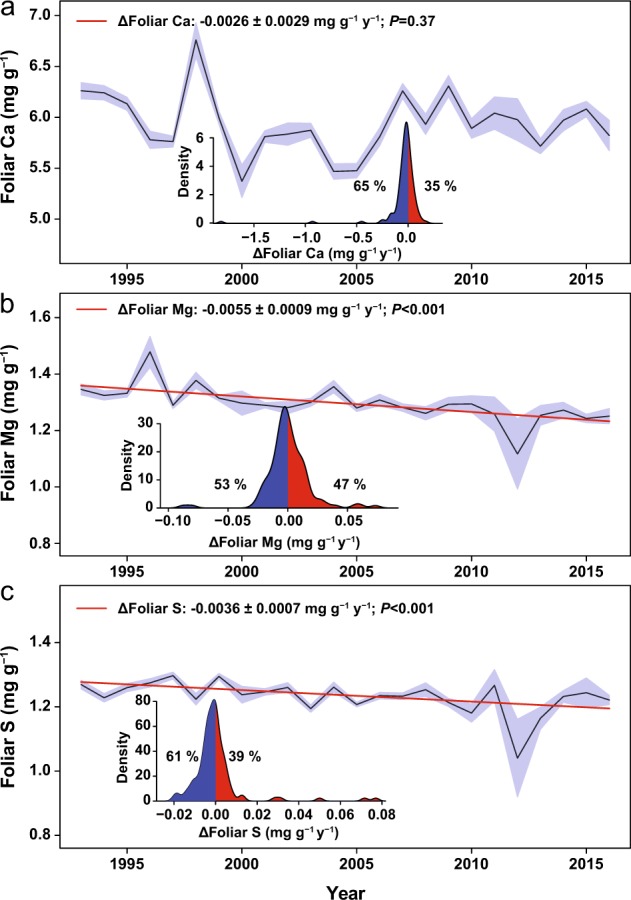
Decreasing tree foliar Ca, Mg and S concentrations. The black lines indicate the average trends, and the shaded areas indicate the standard errors of the average trends. The inset shows the percentages of forests with decreasing and increasing foliar nutrient concentrations. Red and blue indicate forests with increasing and decreasing trends, respectively. All values were adjusted to the same mean to remove forest-specific variability. See Supplementary Table 1 for detailed results of the model lme (foliar variable ∼year, random = ∼1|country/plot/species, data = dades, method = “REML”). Statistically significant trends (in percentage of sites):Ca+: 7.46%,Ca−: 6.45%; Mg+: 7.11%, Mg−: 8.13%; S+: 6.09%, S−: 12.19%.

**Fig. 4:**
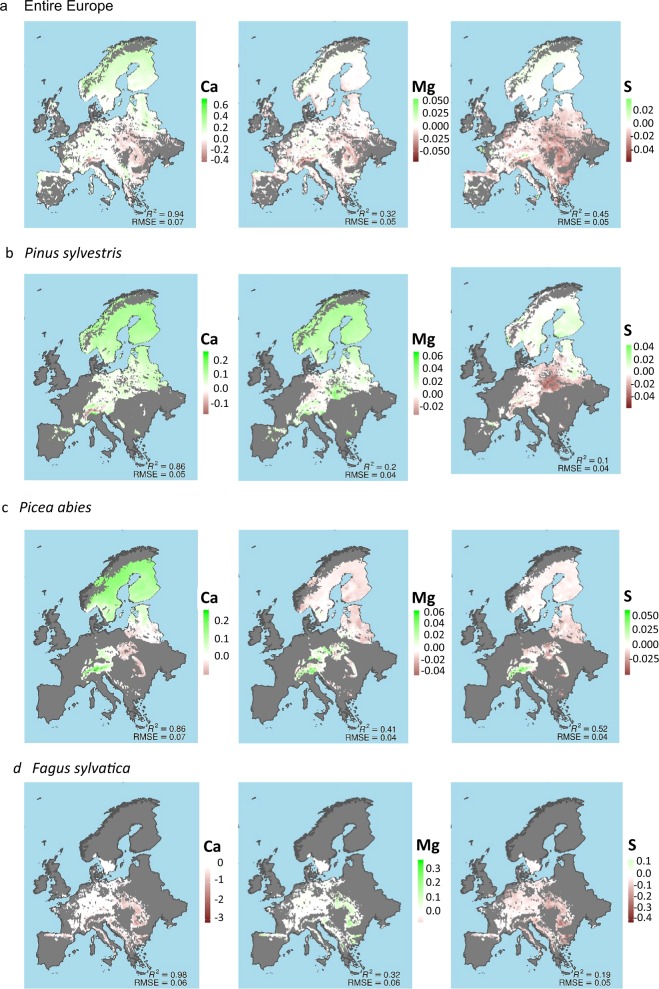
Geographical distribution of the trend in annual rate of variation for Ca, Mg, and S foliar concentration in mg g^−1^. **a** the entire forests, **b** *Pinus sylvestris*, **c** *Picea abies*, and **d** *Fagus sylvatica*. The estimations are based on neural networks using 80% of the trees with more than five measurements for training and 20% for validation. We replicated the process 1000 times and averaged the results.

Foliar N, P and K concentrations decreased in European forests during the last three decades, by 5%, 11% and 8%, respectively (Fig. 1), especially in central and southern Europe (Fig. 2 and Supplementary Fig. 1). An exception is northern Europe where foliar N concentration increased and foliar P concentrations did not change. The foliar N:P ratio increased everywhere by an average of 7% (Fig.1 and Supplementary Fig. 2). Foliar S and Mg concentrations decreased in Europe, by 6% and 7%, respectively (Fig. 3), although foliar S concentrations, as Ca concentrations, increased in northern Europe and decreased in central Europe (Fig. 4 and Supplementary Fig. 3). The trends were not dominated by any extreme values; the effect of the anomalously high or low years for Mg and S concentrations (Fig. 3) was minimised in the mixed model analyses. The results of the analyses after removing years 1996 and 2012 (respectively anomalously high and low) consistently showed that foliar Mg still decreased at a rate of −0.0036 ± 0.0007 mg g^−1^ (*P* < 0.0001)—lower slope than in Fig. 3—and S decreased at a very similar rate as shown in Fig. 3 (−0.0030 ± 0.0007 mg g^−1^, *P* < 0.0001).

**Fig. 5:**
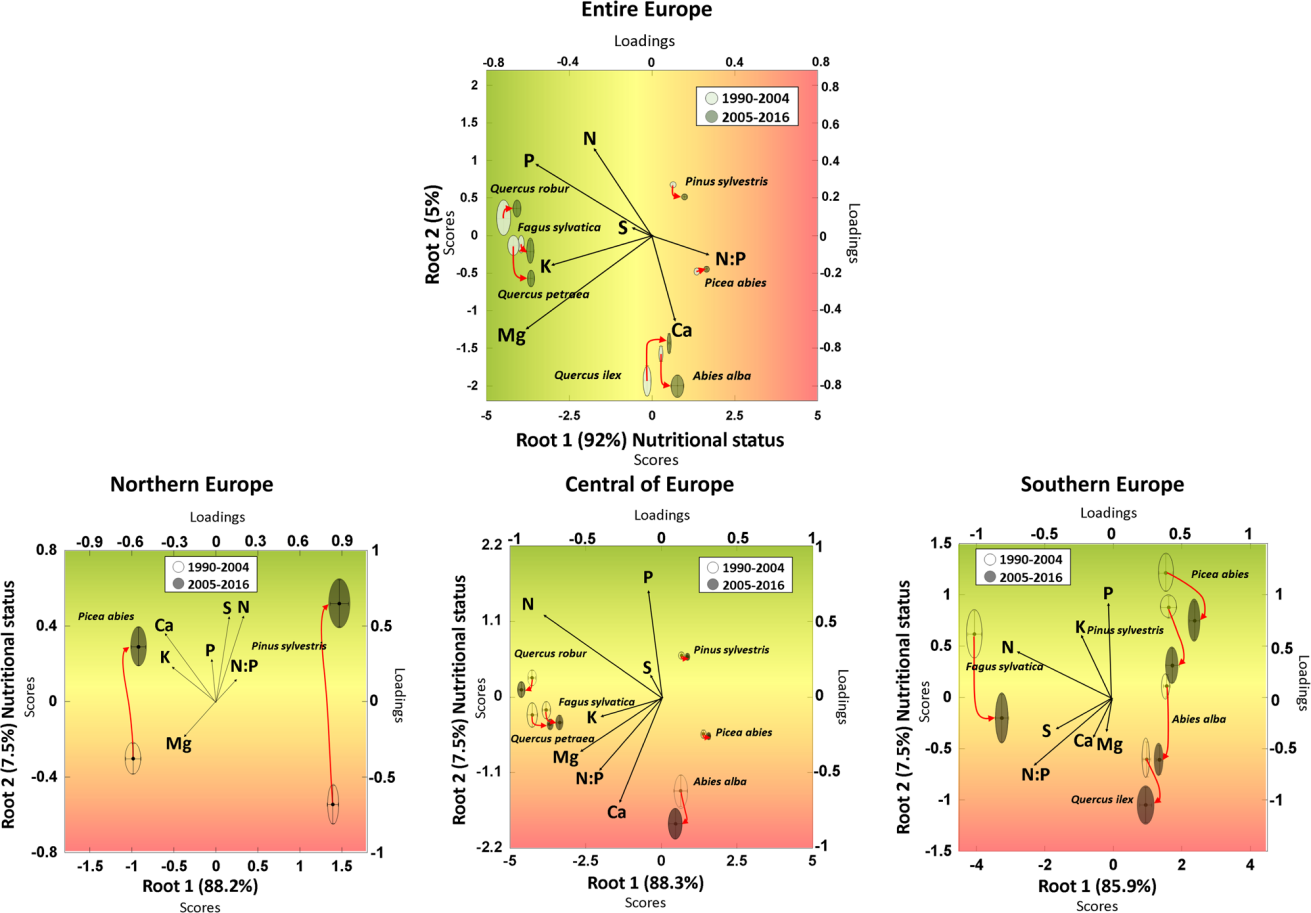
Shifts of the elementome (elemental composition) of European tree species toward lower nutrient concentrations except for northern latitudes. Discriminant analyses of the foliar elemental concentrations and N:P ratios for the seven dominant species, *Pinus sylvestris*, *Picea abies*, *Abies alba*, *Fagus sylvatica*, *Quercus robur*, *Quercus petraea* and *Quercus ilex*, for the entire Europe and for northern, central and southern Europe. All plots compare the data for 1990–2004 with the data for 2005–2016. The circles/ellipses for each species and period depict the mean position and the space occupied by the 95% confidence interval for the scores of each species.

The foliar elemental concentrations followed similar trends in all species (Fig. 2 and Supplementary Figs. 4–9), with few exceptions such as P and Mg increase in *P. sylvestris* at northern latitudes (Figs. 2 and 4). Each species had a distinct foliar elemental composition (elementome), even though the individual trees grew under different environmental conditions and limitations at distinct latitudes (see Fig. 5 and Supplementary Tables 3–10 for a DA and Supplementary Fig. 10 for similar results in a PCA; the DA and the PCA were applied to multi-elemental data space to quantify the ‘elementome’), result consistent with recent studies showing the strong species identity in foliar elemental composition^53^. But despite the observed species identity in their foliar elemental composition, all studied seven species, i.e. *F. sylvatica, P. sylvestris, A. alba, P. abies, Q. ilex, Q. petraea* and *Q. robur*, changed their elemental composition between 1990 and 2016. They shifted their foliar elemental composition along the axis toward decreasing foliar P, K and Mg concentrations and increasing N:P ratio during 2005–2016 relative to 1990–2004. The overall nutritional status of all species thus declined. The regional analysis, though, indicated that this decline did not extend to the northern forests. The foliar elemental composition of the trees at northern latitudes, mostly of *P. sylvestris* and *P. abies*, shifted toward increasing N but also to increasing N:P ratio (Fig. 5b) suggesting a softening of N limitation.

### Possible drivers

**Fig. 6:**
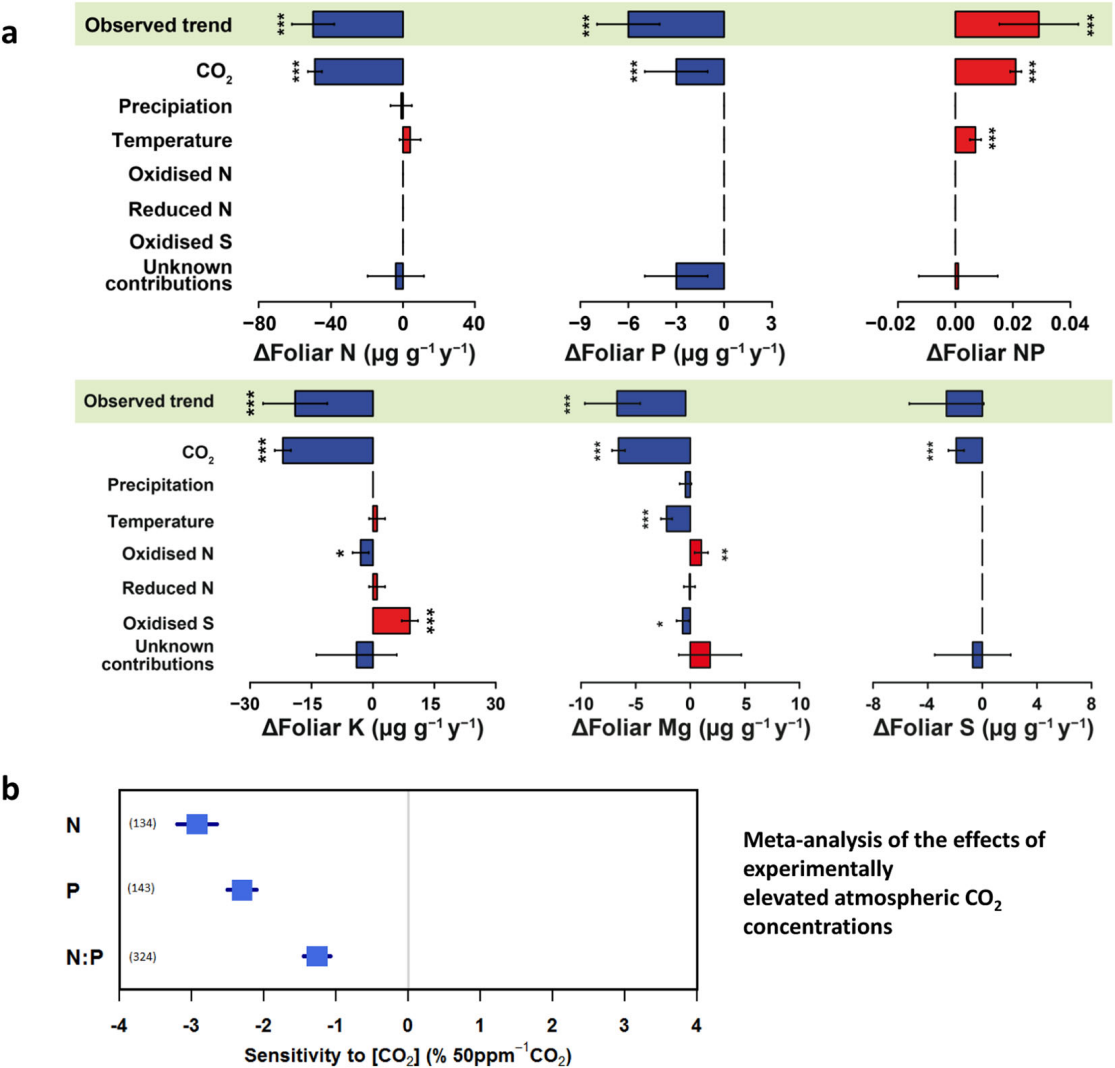
**a** Factors contributing to the decreasing tree foliar N, P, K, S and Mg concentrations and **b** a meta-analysis of the effects of experimentally elevated atmospheric CO_2_ concentrations (using both FACE and OTC methodologies) on the N, P, K, Mg and Ca concentrations and the N:P ratio of green mature leaves for all types of vegetation. **a** Plot of the temporal contribution of the predictor variables on N, P, K, S and Mg concentrations and the N:P ratio (Ca concentration did not change significantly; see Fig. 2). The models (see Supplementary Table 1) suggest that increasing CO_2_ is the main and only contributor to the decreases in N, P, K and S concentrations. The temporal variations of the predictors are shown in Supplementary Fig. 12. Error bars indicate standard errors. Units are ppm for CO_2_, kg ha^−1^ y^−1^ for S and N deposition and °C for temperature. See Methods for information about the methodology used to calculate the contributions. (*), *P* < 0.1; *P < 0.05; **P < 0.01; ***P < 0.001. **b** Meta-analysis of published studies (353, 297 and 684 studies for N and P concentrations and the N:P ratio, respectively). See references in Supplementary material for Fig. 6b.

CO_2_ concentrations during 1990–2016 increased everywhere by ca. 50 ppm, N and S deposition decreased on average by ca. 25% and 65%,respectively, and temperature increased everywhere, especially in the north by almost 1 °C, whereas precipitation increased by ca. 50 mm year^−1^ in the north and decreased by ca. 100 mm year^−1^ in the south (Supplementary Fig. 11). The increase in atmospheric CO_2_ was the only predictor systematically associated with the decreases in nutrient concentrations (Fig. 6). The mixed-model analyses at the level of individual trees produced similar results (Supplementary Table 2). The regional analyses indicated that the increases in some foliar nutrient concentrations in northern Europe were associated with increasingly favourable MATs at northern latitudes (see interactions CO_2_ x MAT in Supplementary Table 1). These mixed-model analyses also included tree growth, which was not selected in any of the explanatory models. The decreases in nutrient concentrations were thus not due to a dilution as a result of individual tree growth, because the increase in the diameter of the tree stems at breast height was never in the most parsimonious models for the driving factors.

We then conducted a meta-analysis of the available literature on open-top chambers (OTC) and FACE (Free-Air Carbon dioxide Enrichment) experiments to test for changes in foliar nutrient concentrations in response to experimentally elevated CO_2_. The decreases in N and P concentrations in response to similar increases of 50 ppm CO_2_ were 3.0% and 2.3% respectively, with the consequent decrease in N:P ratios (Fig. 6b), i.e. both the data from European forests and the data from elevated CO_2_ experiments showed a decreasing effect of increasing CO_2_ on N and P concentration. The data from the experiments showed though lower decreases than those observed in the European forests, especially for P (Fig. 1).

## Discussion

The overall nutritional status of all species declined. The regional analysis, though, indicated that this decline did not extend to the northern forests. A general decline in plant nutritional status has also been reported in other regions such as North America^54^. Herbarium analyses^1,54–57^ also indicated that declines in plant N have already occurred in the last century despite increases in N deposition. Our findings are also consistent with a recent study suggesting a general global pattern of decreasing foliar N concentration ca. 9% over the last 40 years^56^ and with many examples of local to regional decreases in foliar N and P concentrations^24,54^.

Consistently with our results, recent meta-analyses of elevated CO_2_ experiments have found that rising CO_2_ concentrations have led to decreases in N:P ratios in different plant tissues^58^ and woody plants^59^. Deng et al.^58^ hypothesise that the experimental increases in atmospheric CO_2_ concentrations stimulate higher plant uptakes of P than N. However, FACE and OTC experiments are not fully comparable with a progressive increase of atmospheric CO_2_ concentrations in natural conditions where not only CO_2_ concentrations change but also many other factors at the same time. For example, several European regions have become more arid in the last decades, especially in southern Europe^60^ and rises of plant N:P ratios have been reported in response to increasing drought^13,61^. Consistently, we have also observed that MAT has contributed significantly to increase foliar N:P ratios. FACE and OTC, instead, estimate the CO_2_ effects by comparing treatment with control plots with all the other changes affecting equally to both treatment and control plots. In ICP data for European forests some other not controlled factors may have also contributed to decrease more foliar P than N concentration. The frequency and intensity of some forest pests have increased^62,63^, so biotic factors not controlled in this study could have also favoured the P-uptake drop^64^. Moreover, soil P availability tends to decrease through time by natural processes^65^ whereas N availability can continuously be maintained or increase by continuous loads of N deposition and N_2_-fixation. The short-term character of these experiments also does not allow the development of long-term processes, such as the feed-backs due to lower litter quality and the decreases in N and S deposition in recent decades^5^. The experimental decrease may have been smaller also because the experiments test responses to increases in atmospheric CO_2_ concentrations in a less sensitive range of higher CO_2_ concentrations than the actual current range of 360–410 ppm.

The decrease in N deposition was slightly and positively related with foliar Mg and negatively with K concentrations. The decrease in S deposition was slightly and positively related with foliar K and negatively with Mg concentrations. The effects of the shifts in N and S deposition on foliar elemental composition during the studied period were thus weak. Sulfur deposition has dropped in general across Europe since 1980s, but N deposition despite having decreased in general at European scale in the last two decades, has not decreased in all sites, N loads, despite lower, continue being substantial, and, in general, no symptoms of significant recovery of soil status have been observed (Schmitz et al.^5^, and the references therein). N deposition can cause deficiencies in other nutrients than N and nutrient imbalances due to a range of effects, including stimulation of plant growth (dilution effect) and negative effects on tree nutrient acquisition by modifying mycorrhizal associations^24,66^. Increasing mean annual temperature (MAT) also contributed to the decrease in Mg and the increase in Ca and N:P. Ca Moreover, in general, increases in soil pH translate into higher foliar cation concentrations^67–69^.

The higher temperatures at the northern European sites favoured longer growing seasons, biological activity and nutrient up-take, thus accounting for the lower general decreases in foliar nutrient concentrations and even the increase in N. In contrast, the increases in temperature and consequently in drought (decreases in mean annual precipitation (MAP) in southern Europe could account for the decline in soil fertility and capacity of nutrient uptake, all of which contribute to a decrease in foliar nutrient concentration, as in experimental drought studies conducted in Mediterranean forests where mineralisation, soil enzymatic activity and plant growth also decreased, thus leading to a large decrease in aboveground nutrient mineralomasses^9,35,70–72^. A widespread decline in crown condition, disruption of food webs and increased tree mortality with increased drought associated with climate change have also been reported for these southern European forests^60^.

Other foliar chemistry factors and processes not-measured in ICP Forest such as resorption or tree age could also be underlying the observed decrease of foliar nutritional condition. However, the leaves selected for foliar analyses are mature non-senescent leaves and the trees selected for foliar analyses are adult non-senescent trees, and given the frequent long life of trees, the changes in tree age during the studied period, ~25 years as maximum in the individual plots, should not affect much the foliar concentration. Furthermore^73^, reported that foliar N contents and dry weight tended to slightly decrease with age but that this was not the case for N concentrations in *Fagus sylvatica* and *Picea abies* in European forests.

Nutrient impoverishment can have multiple effects on the structure, function and ecosystem services of forests. For example, N and P are fundamental to C assimilation and protein synthesis, so their decreased concentrations could constrain the capacity to take up carbon and the effect of CO_2_ fertilisation in forests^8^. Foliar N:P ratios are negatively correlated with plant net photosynthesis and growth^3^, so the increasing foliar N:P ratios in European forests (Fig. 2) suggested a worsening nutrient imbalance that may partly account for a lower effect of CO_2_ fertilisation^8^. The consequent changes in plant C:N and C:P stoichiometries can also drive ecosystem-level N availability by the effects on litter quality decrease, microbial N immobilisation and mineralisation^19^. The reduction in the availability of N may in turn affect the efficiency of sequestration of C. Increases in foliar C and decreases in N and P are associated with increases in non-structural carbohydrates and carbon-based secondary metabolites^41,74^ and decreases in foliar protein content, thus decreasing the nutritional quality of plants^56,75,76^ for wildlife and livestock.

We thus conclude that foliar concentrations of N, P, K, S and Mg are generally decreasing in European forests. These decreases are generally larger for P than N, so the foliar N:P ratio has increased in most European forests. These trends are mostly associated with increasing atmospheric CO_2_ concentrations that have led to a higher nutrient demand by trees. The soil nutrient supply was probably not always sufficient to meet the growing demands by trees, which could partly explain the deterioration of tree mineral nutrition. These decreasing trends are stronger in southern and central Europe than in northern Europe where the concentrations of some elements are even increasing, all in consonance with the increase in MAT favouring nutrient availability and uptake in northern Europe while hindering them in increasingly dry southern Europe. These nutrient limitations for forest growth should be taken into account by the scientific and environmental management communities to avoid overestimations of forest productivity in response to elevated atmospheric CO_2_ when developing global climate models and projections. The consequences of such pervasive nutrient impoverishment can be key for forest structure, function, health and capacity to provide ecosystem services.

## Methods

### Data acquisition

We used foliar and growth data of the International Co-operative Programme on Assessment and Monitoring of Air Pollution Effects on Forests operating under the UNECE Convention on Long-range Transboundary Air Pollution (CLRTAP) (ICP Forests). Activities under ICP Forests are conducted in 42 states using harmonised sampling and analysis following the manuals on Sampling and Analysis of needles and leaves—Part XII: http://www.icp-forests.org/pdf/manual/2016/ICP_Manual_2017_01_part12.pdf and Tree growth—Part V: (http://www.icp-forests.org/pdf/manual/2016/ICP_Manual_2016_01_part05.pdf). In this study, we have gathered all the available data of foliar N, P, K, S, Ca and Mg concentrations in annual series. We have used data from 28 countries with a total of 528 different plots and with 506 of these plots with the canopy clearly dominated by one tree species, 21 co-dominated by two tree species, and 1 plot co-dominated by three tree species.

Foliar samples were taken at least biannually at the intensive forest monitoring plots (Level II) from the living crown of the dominant canopy layer providing information on the nutrient status of one or more species per plot. The analysis was done for 1000 needles or 100 leaves covering a range of elements (for detailed information please refer to the ICP Forest Manual). Briefly, in each plot a minimum of five dominant trees were randomly selected avoiding the trees used in crown assessment, so to avoid crown damage of these trees. A composite sample by species was made by mixing equal quantities of each sample per plot after drying. Sampled leaves were mature current year leaves or needles. Only in the case of evergreens such as *Q. ilex* both mature (non-senescent) one- and second-year leaves were sampled. The sample analyses procedure was based on homogenised methods, basically direct C and N determination by elemental-analyser (Kjeldhal method was also allowed for N determination), whereas for the other elements the most common procedure was based on acid digestion with nitric acid or nitric acid mixtures (43–46%) followed by wet ashing (40%) and posterior determination, mostly by inductively coupled plasma coupled to atomic emission spectrometry (ICP-AES). Quality assurance and assessment of the analytical process was controlled by the regular organisation of Inter-laboratory Comparisons by the Forest Foliar Co-ordinating Centre. The laboratory results were considered of sufficient quality when the laboratory receives a qualification for the concerning parameter(s) after participation in the inter-laboratory comparisons. The growth survey assessed the diameter at breast height (dbh) for the dominant trees at a five or ten yearly interval. Additionally, permanent girth bands provided annual data of dbh.

For atmospheric CO_2_ concentration, we used monthly data from the Mauna Loa Observatory, available online (http://scrippsco2.ucsd.edu/data/atmospheric_co2/) and provided by the Scripps Institution of Oceanography (Scripps CO_2_ programme). We calculated the annual averages to use in our statistical analyses. We obtained the meteorological-climate data from CRU TS v3.25 of the Climatic Research Unit^77^. Annual data for N (NO_3_^−^ + NH_4_^+^) and S (SO_4_^−^) atmospheric deposition were extracted from the European Monitoring and Evaluation Programme (EMEP) with a spatial resolution of 0.1 × 0.1° for longitude and latitude, and the MSC-W chemical transport model developed to estimate regional atmospheric dispersion and deposition of acidifying and eutrophying compounds of N and S over Europe.

### Statistics and reproducibility

First, we used time series of observations of foliar N, P, K, S, Ca and Mg concentrations at 410 European sites (Supplementary Fig. 12) for the last three decades (1990–2016) to investigate their temporal trends using mixed models, where year was the fixed covariate and site-species was the random factor. Second, we repeated the analyses separately for northern, central and southern European forests (separated by parallels at 46° and 58° North; Supplementary Fig. 1b). Third, we repeated the previous two analyses for each of the most abundant species: *F. sylvatica*, *P. sylvestris*, *and P. abies*. Fourth, we predicted the rate of changes in foliar elemental concentration across Europe as a function of MAP, MAT and nutrient deposition rate of change using neural networks. We calculated the rate of changes of the foliar elements for each tree with more than five measurements and the rate of change of the MAP, MAT and nutrient deposition for the same period and location using Theil-Sen robust regressions implemented in mblm R package^71^. Then, we used 80% of the estimated rates to train the neural networks and 20% for validation using keras^78^ in R^79^ with TensorFlow^80^ backend. The neural networks had two hidden layers with 128 units each with rectified linear activation functions. We repeated the process 1000 times making predicted maps and averaging the results. Finally we masked the pixels with no forest^81^ and for species specific models, we also applied a mask with the distribution maps^82–84^. Fifth, we used multivariate analyses, including a discriminant analysis (DA) and a principal component analysis (PCA) of all nutrient variables to analyse the shifts in the elementome^53,85,86^ for each species for the entire Europe and each of the three latitudes.

We explored which environmental factors could better explain the observed changes in foliar elemental concentrations. To do so, we estimated the temporal contributions of the predictor variables to the trends of foliar N, P and K concentrations and the N:P ratio following the methodology established by Fernandez-Martinez et al.^41,87^. We first fitted one model for each foliar nutrient using a generalized linear mixed model (GLMM), with the species and experimental plot as random factors, using the lme function from the nlme R package^88^ and an autoregressive and moving-average (ARMA) (*p* = 1, *q* = 0) temporal autocorrelation structure using the corCAR1 function. We then fitted the full (saturated) models for each foliar nutrient as a function of atmospheric CO_2_, MAT and MAP and N (oxidised and reduced) and S deposition. We also included the interactions between mean site values and their temporal anomalies to account for different effects of, for example, increasing temperature (annual anomalies) depending on the average temperature of the site (e.g. decreasing temperature may have a positive effect in warm climates but a negative effect in cold climates). We also included the first-order interactions between atmospheric CO_2_, climate and variables of atmospheric deposition. We then removed non-significant terms from the full models until obtaining the final model (containing only significant terms) using stepwise backward-forward selection. The amount of the variance explained by the models was assessed using the r.squared GLMM function in R (MuMInpackage: Barton^89^) following the method of Nakagawa and Schielzeth)^90^.

We next used the final models (lme models explained above) to identify the drivers contributing to the changes in foliar nutrient concentrations using the *TempCont* R package. We first calculated the trend (mean ± standard error of the mean) of a foliar nutrient concentration using raw data with GLMMs with an ARMA (*p* = 1, *q* = 0) structure to account for temporal autocorrelation. We then calculated the trends of foliar nutrient concentrations predicted by the final model (containing all significant predictors) and the trends of the foliar nutrient concentrations predicted by the same model but sequentially maintaining the temporally varying predictors constant (e.g. temporal anomalies in MAT were held constant using the median for each site, but all other predictors varied based on the observations). The difference between the prediction of the final model and the prediction of the model when one predictor was controlled was assumed to be the contribution of that predictor to the temporal change in foliar nutrient concentrations. The differences between the sum of all temporal contributions and the observed trend of a foliar nutrient were considered as unknown temporal contributions. All errors were calculated using error propagation.

We finally conducted a meta-analysis of published experimental data for the environmental factor that was best correlated with nutrient depletion: CO_2_ fertilisation. We used the keywords: atmospheric CO_2_, C:N, CO_2_ fertilisation, C:P, decrease, dilution, FACE, foliar, increase, leaf, needle, nitrogen, N:P, phosphorus, photosynthetic tissues, rise, and stoichiometry in our web search in Web of Science and google scholar between 1988 and 2018. We gathered the available studies that tested for the effects of elevated atmospheric CO_2_ concentrations (both using FACE and OTC methodologies) on N and P concentrations and the N:P ratio of green mature leaves for all types of vegetation (353, 297 and 684 studies, respectively). The list of the published articles considered appears into supplementary Material. The R metafor (v. 2.0-0) and forest plot (v. 1.7.2) packages were used for the analyses as described in Hedges et al.^91^. We calculated the response ratio (lnRR) as ln (Xi/Xn) = lnXi - lnXn, where Xi and Xn are the values of each chemical compound in leaf tissue observation before and after the treatment respectively. The sampling variance for each lnRR was calculated as ln[(1/ni) × (Si/Xi)^2^ + (1/nn) × (Sn/Xn)^2^], where ni, nn, Si, Sn, Xi and Xn are the post-treatment and control sample sizes, standard deviations, and mean response values, respectively. The sensitivity was evaluated per ppm of CO_2_ added in the treatment. We thereafter standardised the effect size to an increase of 50 ppm since atmospheric CO_2_ concentrations increased everywhere by ca. 50 ppm during 1990–2016.

### Reporting summary

Further information on research design is available in the Nature Research Reporting Summary linked to this article.

## Supplementary information

Supplementary Information

Reporting Summary

## Data Availability

The datasets generated during and/or analysed during the current study are available from the corresponding author, Prof. Josep Penuelas.

## Code availability

All codes are available upon request to the corresponding author, Professor Josep Penuelas.
