# Supplementary material for: Increasing atmospheric CO2 concentrations correlate with declining nutritional status of European forests
Source: Commun Biol. 2020 Mar 13;3:125. doi: 10.1038/s42003-020-0839-y (PMC7070084; doi:10.1038/s42003-020-0839-y)
Supplement: Supplementary file 1 — Supplementary Information [file 42003_2020_839_MOESM1_ESM.pdf]

Supplementary information

Supplementary Figure 1. **Time series of the tree foliar N, P, and K concentrations for northern, central and southern Europe**. See Table S1 for detailed results of the model lme (foliar variable ~ year, random=~1|country/plot/species, data=dades, method="REML").

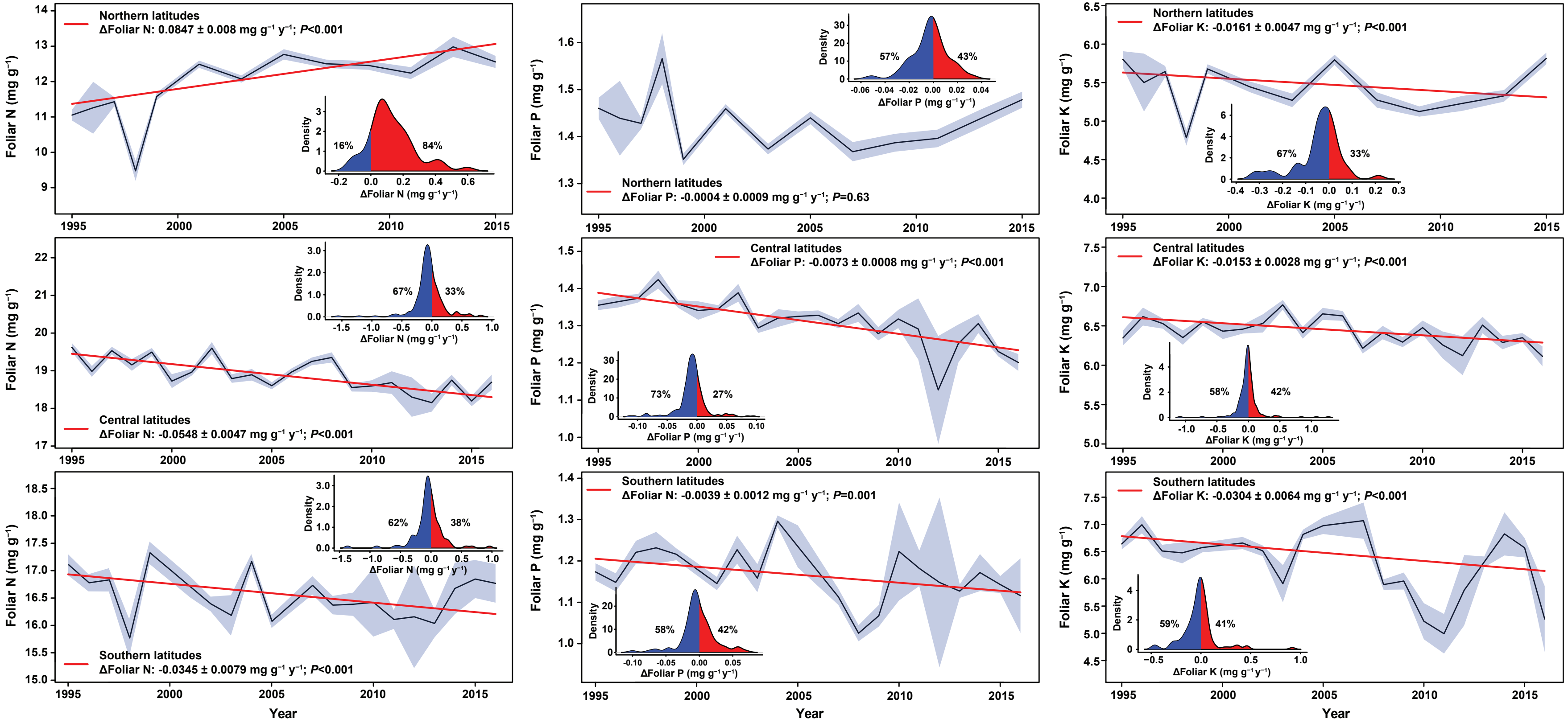

Northern latitudes
ΔFoliar N: 0.0847 ± 0.008 mg g^{-1} y^{-1}; *P*<0.001
Foliar N (mg g^{-1})
16%
84%
Density
ΔFoliar N (mg g^{-1} y^{-1})
Northern latitudes
ΔFoliar P: -0.0004 ± 0.0009 mg g^{-1} y^{-1}; *P*=0.63
Foliar P (mg g^{-1})
57%
43%
Density
ΔFoliar P (mg g^{-1} y^{-1})
Northern latitudes
ΔFoliar K: -0.0161 ± 0.0047 mg g^{-1} y^{-1}; *P*<0.001
Foliar K (mg g^{-1})
67%
33%
Density
ΔFoliar K (mg g^{-1} y^{-1})
Central latitudes
ΔFoliar N: -0.0548 ± 0.0047 mg g^{-1} y^{-1}; *P*<0.001
Foliar N (mg g^{-1})
67%
33%
Density
ΔFoliar N (mg g^{-1} y^{-1})
Central latitudes
ΔFoliar P: -0.0073 ± 0.0008 mg g^{-1} y^{-1}; *P*<0.001
Foliar P (mg g^{-1})
73%
27%
Density
ΔFoliar P (mg g^{-1} y^{-1})
Central latitudes
ΔFoliar K: -0.0153 ± 0.0028 mg g^{-1} y^{-1}; *P*<0.001
Foliar K (mg g^{-1})
58%
42%
Density
ΔFoliar K (mg g^{-1} y^{-1})
Southern latitudes
ΔFoliar N: -0.0345 ± 0.0079 mg g^{-1} y^{-1}; *P*<0.001
Foliar N (mg g^{-1})
62%
38%
Density
ΔFoliar N (mg g^{-1} y^{-1})
Southern latitudes
ΔFoliar N: -0.0039 ± 0.0012 mg g^{-1} y^{-1}; *P*=0.001
Foliar P (mg g^{-1})
58%
42%
Density
ΔFoliar P (mg g^{-1} y^{-1})
Southern latitudes
ΔFoliar K: -0.0304 ± 0.0064 mg g^{-1} y^{-1}; *P*<0.001
Foliar K (mg g^{-1})
59%
41%
Density
ΔFoliar K (mg g^{-1} y^{-1})
1995
2000
2005
2010
2015
Year

Supplementary Figure 2. a) **Time series of the tree foliar N:P ratio in northern (a), central (b) and southern (c) Europe**. See Table S1 for detailed results of the model lme (foliar variable ~ year, random=~1|country/plot/species, data=dades, method="REML"). d) **Geographical distribution of the annual rate of variation for N:P**. The pixel estimations are based on Neural Networks using 80% of the trees with more than 5 measurements for training and 20% for validation. We replicated the process 1000 times and averaged the results.

a

b

c

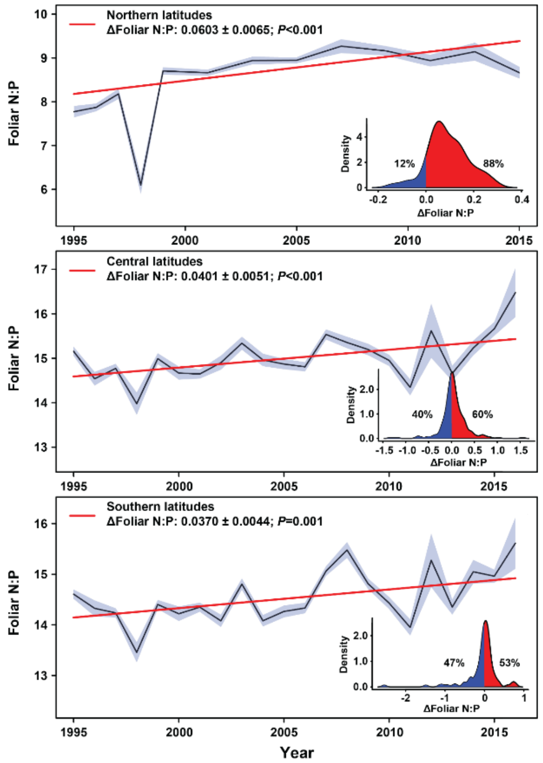

d

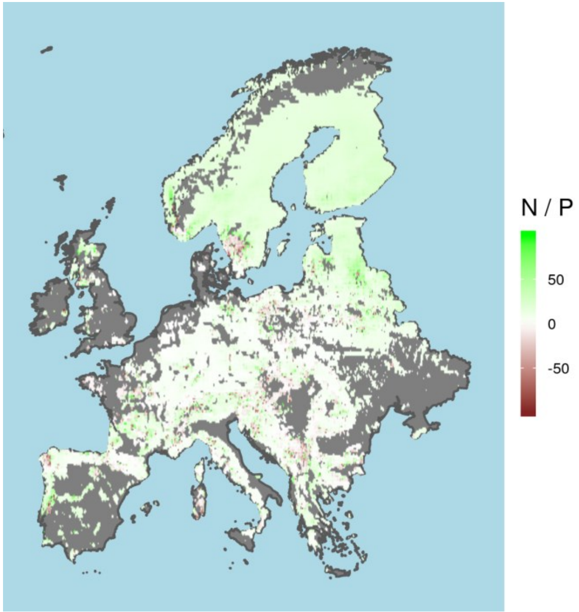

Supplementary Figure 3. **Time series of the tree foliar Ca Mg and S concentrations**. See Table S1 for detailed results of the model lme (foliar variable ~ year, random=~1|country/plot/species, data=dades, method="REML")

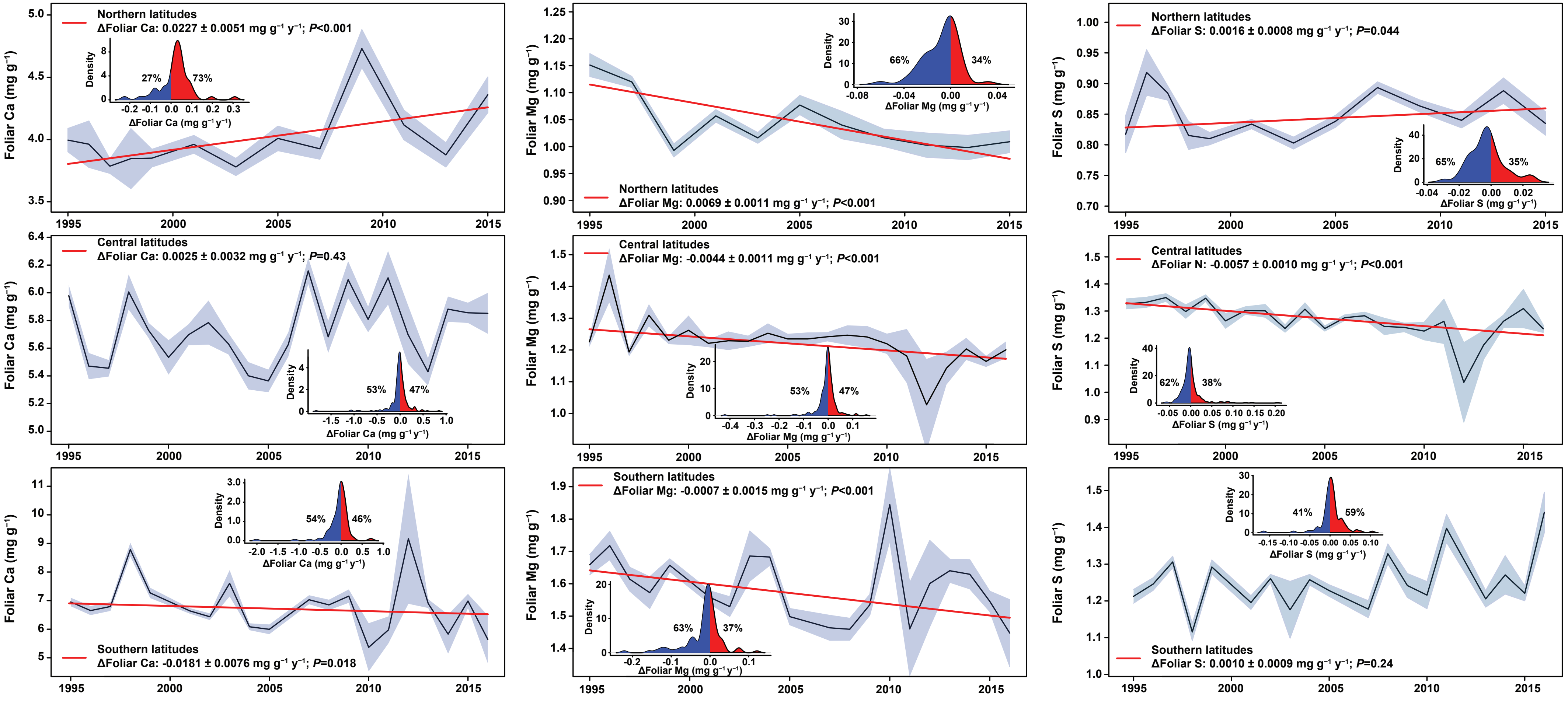

Northern latitudes
ΔFoliar Ca: 0.0227 ± 0.0051 mg g⁻¹ y⁻¹; P<0.001
Northern latitudes
ΔFoliar Mg: 0.0069 ± 0.0011 mg g⁻¹ y⁻¹; P<0.001
Northern latitudes
ΔFoliar S: 0.0016 ± 0.0008 mg g⁻¹ y⁻¹; P=0.044
Central latitudes
ΔFoliar Ca: 0.0025 ± 0.0032 mg g⁻¹ y⁻¹; P=0.43
Central latitudes
ΔFoliar Mg: -0.0044 ± 0.0011 mg g⁻¹ y⁻¹; P<0.001
Central latitudes
ΔFoliar N: -0.0057 ± 0.0010 mg g⁻¹ y⁻¹; P<0.001
Southern latitudes
ΔFoliar Ca: -0.0181 ± 0.0076 mg g⁻¹ y⁻¹; P=0.018
Southern latitudes
ΔFoliar Mg: -0.0007 ± 0.0015 mg g⁻¹ y⁻¹; P<0.001
Southern latitudes
ΔFoliar S: 0.0010 ± 0.0009 mg g⁻¹ y⁻¹; P=0.24
Foliar Ca (mg g⁻¹)
Foliar Mg (mg g⁻¹)
Foliar S (mg g⁻¹)
Density
ΔFoliar Ca (mg g⁻¹ y⁻¹)
ΔFoliar Mg (mg g⁻¹ y⁻¹)
ΔFoliar S (mg g⁻¹ y⁻¹)
27% 73%
66% 34%
65% 35%
53% 47%
53% 47%
62% 38%
54% 46%
63% 37%
41% 59%
1995 2000 2005 2010 2015

Supplementary Figure 4. **Trends of tree foliar N, P, K, Ca, S and Mg concentrations and N:P ratio in *Pinus sylvestris.*** The shaded areas indicate the standard errors of the average trends. All values were adjusted to the same mean to remove forest-specific variability. See Table S1 for detailed results of the model lme (foliar variable ~ year, random=~1|country/plot/species, data=dades, method="REML").

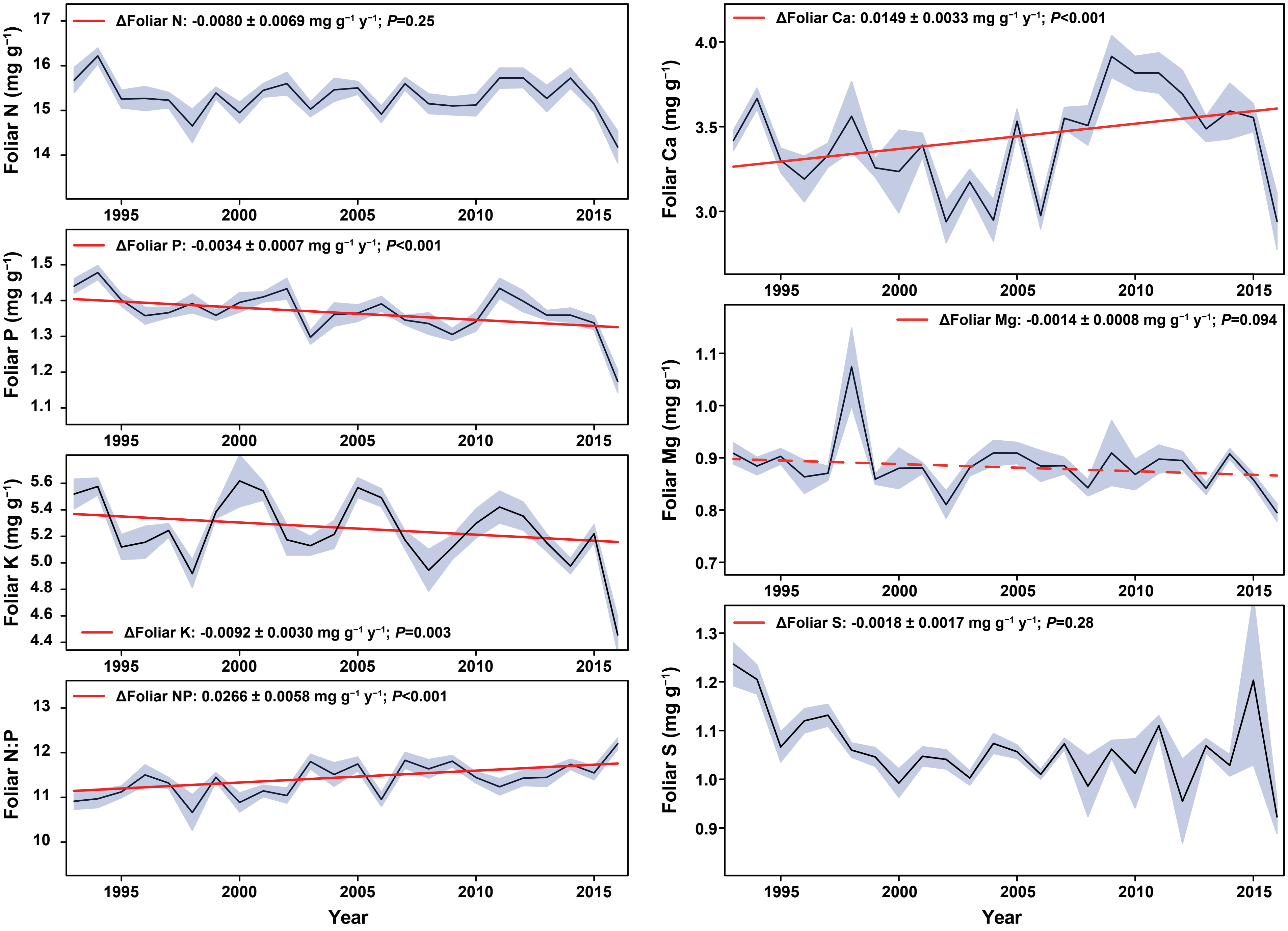

ΔFoliar N: -0.0080 ± 0.0069 mg g⁻¹ y⁻¹; P=0.25
Foliar N (mg g⁻¹)
ΔFoliar P: -0.0034 ± 0.0007 mg g⁻¹ y⁻¹; P<0.001
Foliar P (mg g⁻¹)
ΔFoliar K: -0.0092 ± 0.0030 mg g⁻¹ y⁻¹; P=0.003
Foliar K (mg g⁻¹)
ΔFoliar NP: 0.0266 ± 0.0058 mg g⁻¹ y⁻¹; P<0.001
Foliar N:P
ΔFoliar Ca: 0.0149 ± 0.0033 mg g⁻¹ y⁻¹; P<0.001
Foliar Ca (mg g⁻¹)
ΔFoliar Mg: -0.0014 ± 0.0008 mg g⁻¹ y⁻¹; P=0.094
Foliar Mg (mg g⁻¹)
ΔFoliar S: -0.0018 ± 0.0017 mg g⁻¹ y⁻¹; P=0.28
Foliar S (mg g⁻¹)
Year

Supplementary Figure 5. **Trends of tree foliar N, P, K, Ca, S and Mg concentrations and N:P ratio in *Picea abies.*** The shaded areas indicate the standard errors of the average trends. All values were adjusted to the same mean to remove forest-specific variability. See Table S1 for detailed results of the model lme (foliar variable ~ year, random=~1|country/plot/species, data=dades, method="REML").

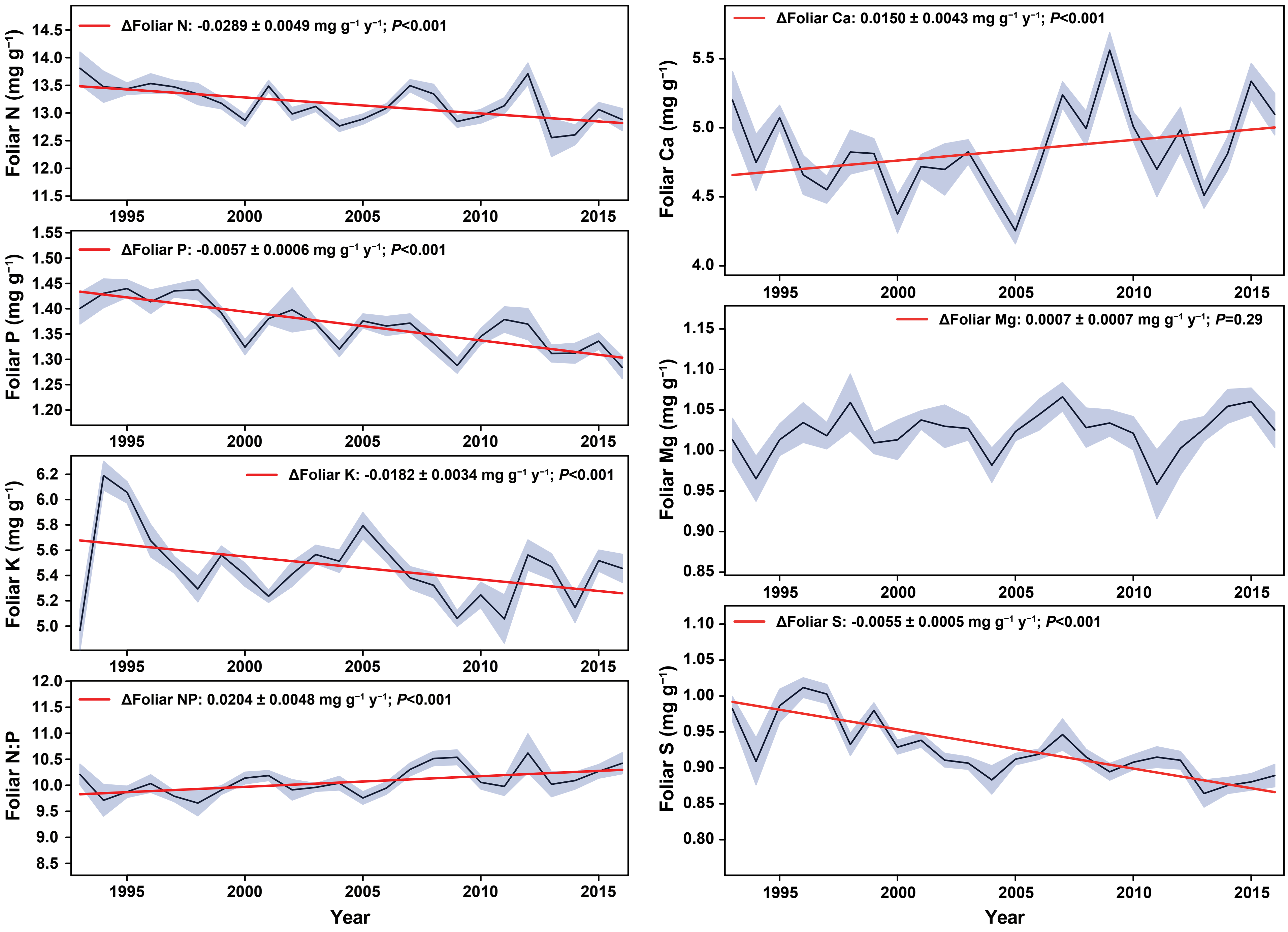

ΔFoliar N: -0.0289 ± 0.0049 mg g⁻¹ y⁻¹; P<0.001
Foliar N (mg g⁻¹)
ΔFoliar P: -0.0057 ± 0.0006 mg g⁻¹ y⁻¹; P<0.001
Foliar P (mg g⁻¹)
ΔFoliar K: -0.0182 ± 0.0034 mg g⁻¹ y⁻¹; P<0.001
Foliar K (mg g⁻¹)
ΔFoliar NP: 0.0204 ± 0.0048 mg g⁻¹ y⁻¹; P<0.001
Foliar N:P
ΔFoliar Ca: 0.0150 ± 0.0043 mg g⁻¹ y⁻¹; P<0.001
Foliar Ca (mg g⁻¹)
ΔFoliar Mg: 0.0007 ± 0.0007 mg g⁻¹ y⁻¹; P=0.29
Foliar Mg (mg g⁻¹)
ΔFoliar S: -0.0055 ± 0.0005 mg g⁻¹ y⁻¹; P<0.001
Foliar S (mg g⁻¹)
Year
Year

Supplementary Figure 6. **Trends of tree foliar N, P, K, Ca, S and Mg concentrations and N:P ratio in *Fagus sylvatica*.** The shaded areas indicate the standard errors of the average trends. All values were adjusted to the same mean to remove forest-specific variability. See Table S1 for detailed results of the model lme (foliar variable ~ year, random=~1|country/plot/species, data=dades, method="REML").

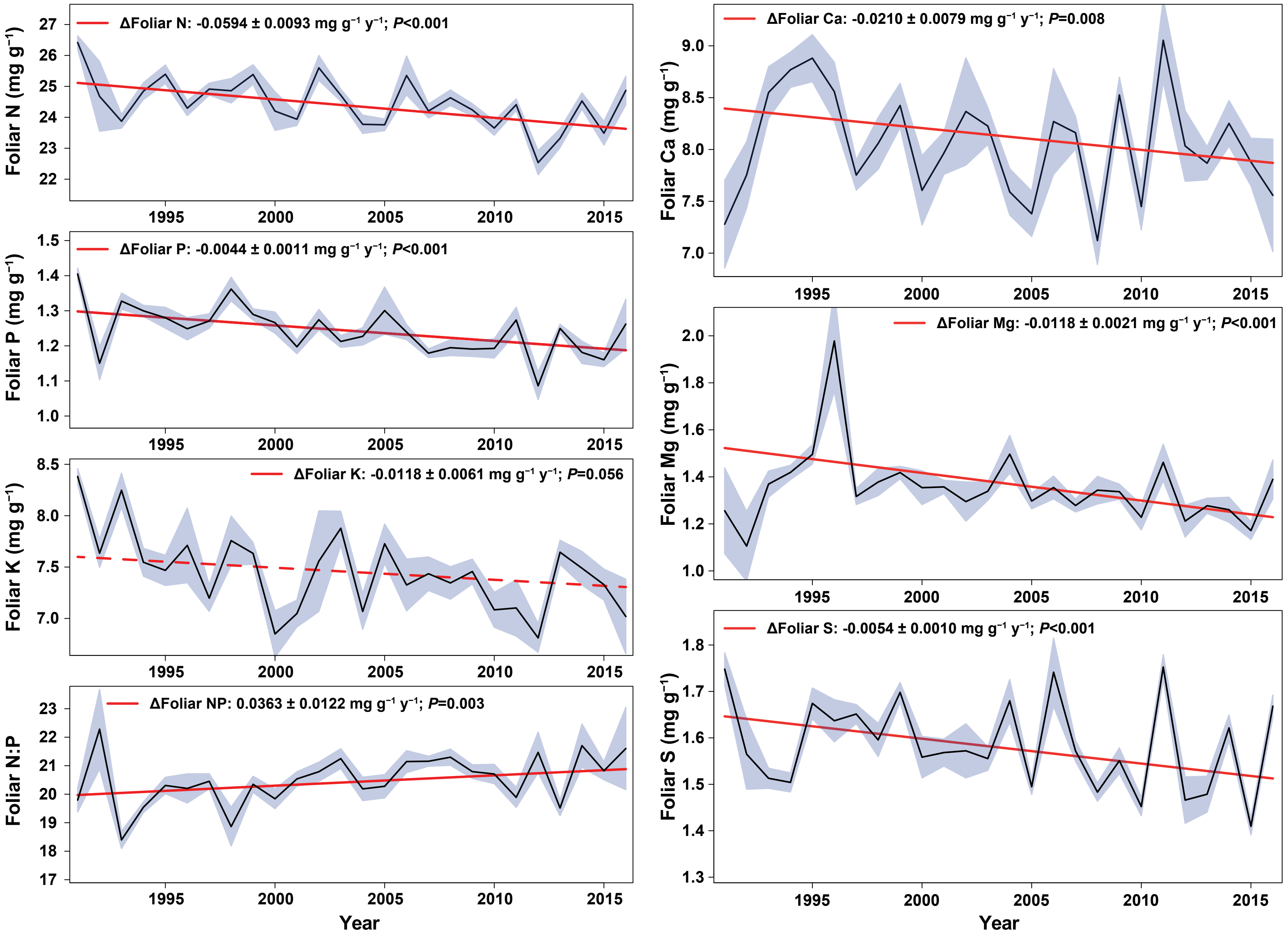

ΔFoliar N: -0.0594 ± 0.0093 mg g⁻¹ y⁻¹; P<0.001
Foliar N (mg g⁻¹)
ΔFoliar P: -0.0044 ± 0.0011 mg g⁻¹ y⁻¹; P<0.001
Foliar P (mg g⁻¹)
ΔFoliar K: -0.0118 ± 0.0061 mg g⁻¹ y⁻¹; P=0.056
Foliar K (mg g⁻¹)
ΔFoliar NP: 0.0363 ± 0.0122 mg g⁻¹ y⁻¹; P=0.003
Foliar N:P
ΔFoliar Ca: -0.0210 ± 0.0079 mg g⁻¹ y⁻¹; P=0.008
Foliar Ca (mg g⁻¹)
ΔFoliar Mg: -0.0118 ± 0.0021 mg g⁻¹ y⁻¹; P<0.001
Foliar Mg (mg g⁻¹)
ΔFoliar S: -0.0054 ± 0.0010 mg g⁻¹ y⁻¹; P<0.001
Foliar S (mg g⁻¹)
Year
Year

Supplementary Figure 7. **Trends of tree foliar N, P, and K concentrations and N:P ratio in *Pinus sylvestris* in northern, central and southern Europe.** The shaded areas indicate the standard errors of the average trends. All values were adjusted to the same mean to remove forest-specific variability. See Table S1 for detailed results of the model lme (foliar variable ~ year, random=~1|country/plot/species, data=dades, method="REML").

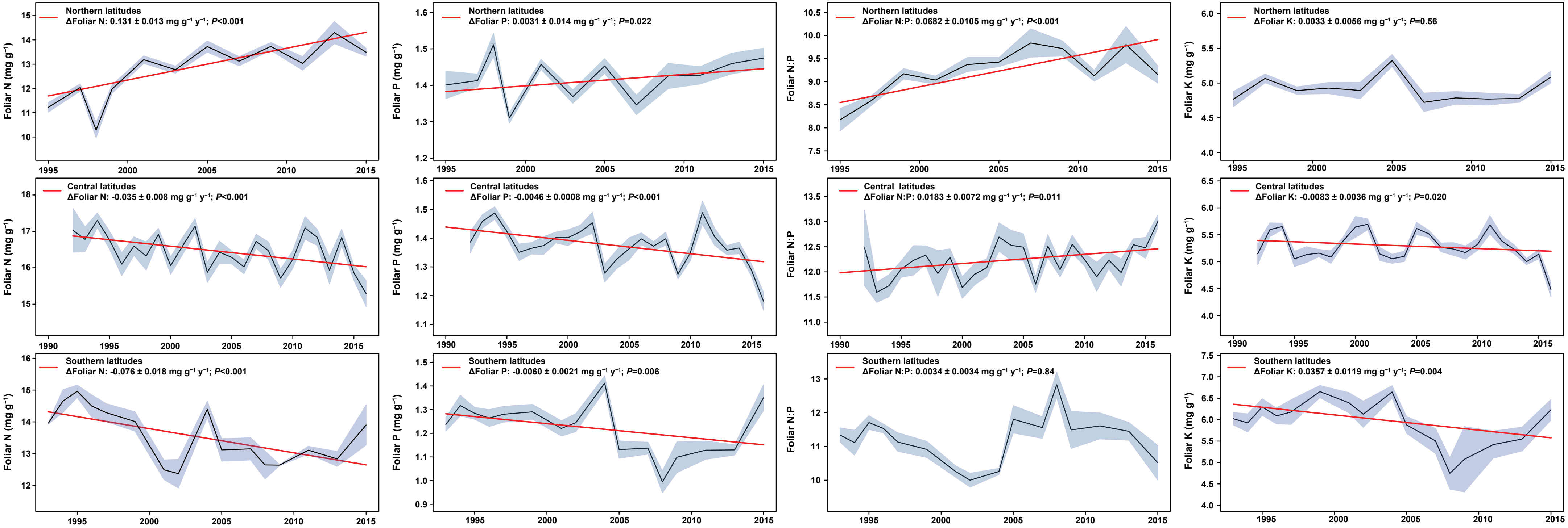

Northern latitudes
ΔFoliar N: 0.131 ± 0.013 mg g⁻¹ y⁻¹; P<0.001
Northern latitudes
ΔFoliar P: 0.0031 ± 0.014 mg g⁻¹ y⁻¹; P=0.022
Northern latitudes
ΔFoliar N:P: 0.0682 ± 0.0105 mg g⁻¹ y⁻¹; P<0.001
Northern latitudes
ΔFoliar K: 0.0033 ± 0.0056 mg g⁻¹ y⁻¹; P=0.56
Central latitudes
ΔFoliar N: -0.035 ± 0.008 mg g⁻¹ y⁻¹; P<0.001
Central latitudes
ΔFoliar P: -0.0046 ± 0.0008 mg g⁻¹ y⁻¹; P<0.001
Central latitudes
ΔFoliar N:P: 0.0183 ± 0.0072 mg g⁻¹ y⁻¹; P=0.011
Central latitudes
ΔFoliar K: -0.0083 ± 0.0036 mg g⁻¹ y⁻¹; P=0.020
Southern latitudes
ΔFoliar N: -0.076 ± 0.018 mg g⁻¹ y⁻¹; P<0.001
Southern latitudes
ΔFoliar P: -0.0060 ± 0.0021 mg g⁻¹ y⁻¹; P=0.006
Southern latitudes
ΔFoliar N:P: 0.0034 ± 0.0034 mg g⁻¹ y⁻¹; P=0.84
Southern latitudes
ΔFoliar K: 0.0357 ± 0.0119 mg g⁻¹ y⁻¹; P=0.004
Foliar N (mg g⁻¹)
Foliar P (mg g⁻¹)
Foliar N:P
Foliar K (mg g⁻¹)

Supplementary Figure 8. **Trends of tree foliar N, P, and K concentrations and N:P ratio in *Picea abies* in northern, central and southern Europe.** The shaded areas indicate the standard errors of the average trends. All values were adjusted to the same mean to remove forest-specific variability. See Table S1 for detailed results of the model lme (foliar variable ~ year, random=~1|country/plot/species, data=dades, method="REML").

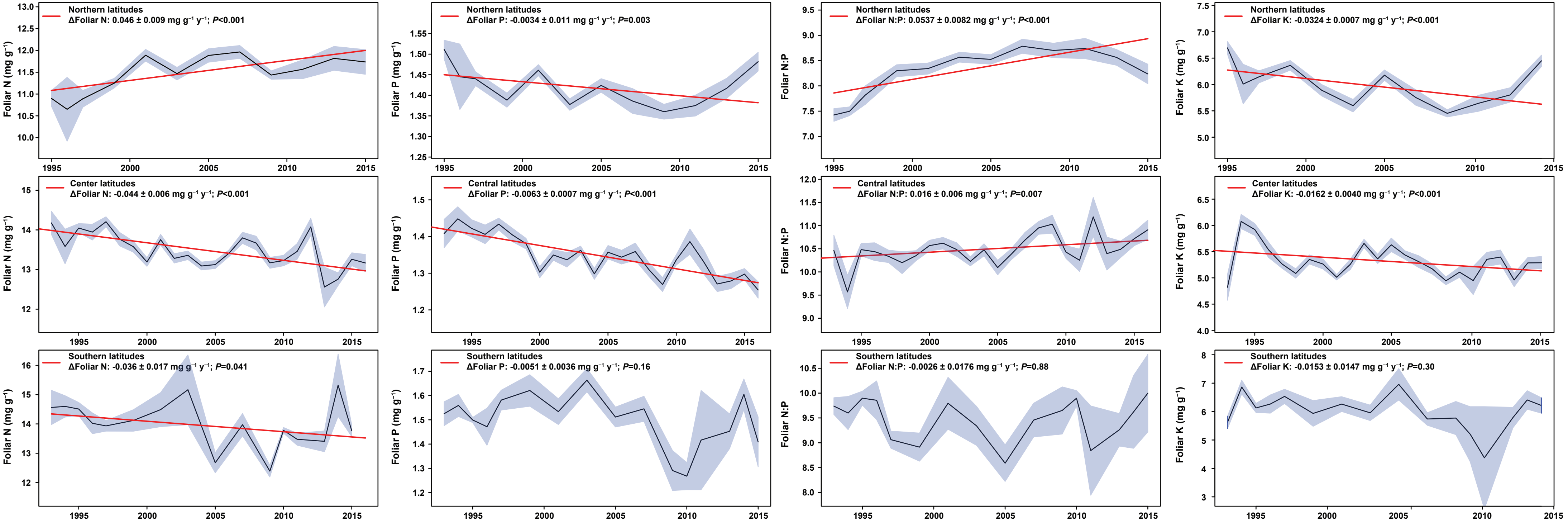
Northern latitudes
ΔFoliar N: 0.046 ± 0.009 mg g⁻¹ y⁻¹; P<0.001
Foliar N (mg g⁻¹)
Northern latitudes
ΔFoliar P: -0.0034 ± 0.011 mg g⁻¹ y⁻¹; P=0.003
Foliar P (mg g⁻¹)
Northern latitudes
ΔFoliar N:P: 0.0537 ± 0.0082 mg g⁻¹ y⁻¹; P<0.001
Foliar N:P
Northern latitudes
ΔFoliar K: -0.0324 ± 0.0007 mg g⁻¹ y⁻¹; P<0.001
Foliar K (mg g⁻¹)
Center latitudes
ΔFoliar N: -0.044 ± 0.006 mg g⁻¹ y⁻¹; P<0.001
Foliar N (mg g⁻¹)
Central latitudes
ΔFoliar P: -0.0063 ± 0.0007 mg g⁻¹ y⁻¹; P<0.001
Foliar P (mg g⁻¹)
Central latitudes
ΔFoliar N:P: 0.016 ± 0.006 mg g⁻¹ y⁻¹; P=0.007
Foliar N:P
Center latitudes
ΔFoliar K: -0.0162 ± 0.0040 mg g⁻¹ y⁻¹; P<0.001
Foliar K (mg g⁻¹)
Southern latitudes
ΔFoliar N: -0.036 ± 0.017 mg g⁻¹ y⁻¹; P=0.041
Foliar N (mg g⁻¹)
Southern latitudes
ΔFoliar P: -0.0051 ± 0.0036 mg g⁻¹ y⁻¹; P=0.16
Foliar P (mg g⁻¹)
Southern latitudes
ΔFoliar N:P: -0.0026 ± 0.0176 mg g⁻¹ y⁻¹; P=0.88
Foliar N:P
Southern latitudes
ΔFoliar K: -0.0153 ± 0.0147 mg g⁻¹ y⁻¹; P=0.30
Foliar K (mg g⁻¹)
1995
2000
2005
2010
2015

Supplementary Figure 9. **Trends of tree foliar N, P, K and Mg concentrations and N:P ratio in *Fagus sylvatica* in northern, central and southern Europe.** The shaded areas indicate the standard errors of the average trends. All values were adjusted to the same mean to remove forest-specific variability. See Table S1 for detailed results of the model lme(foliar variable ~ year, random=~1|country/plot/species, data=dades, method="REML").

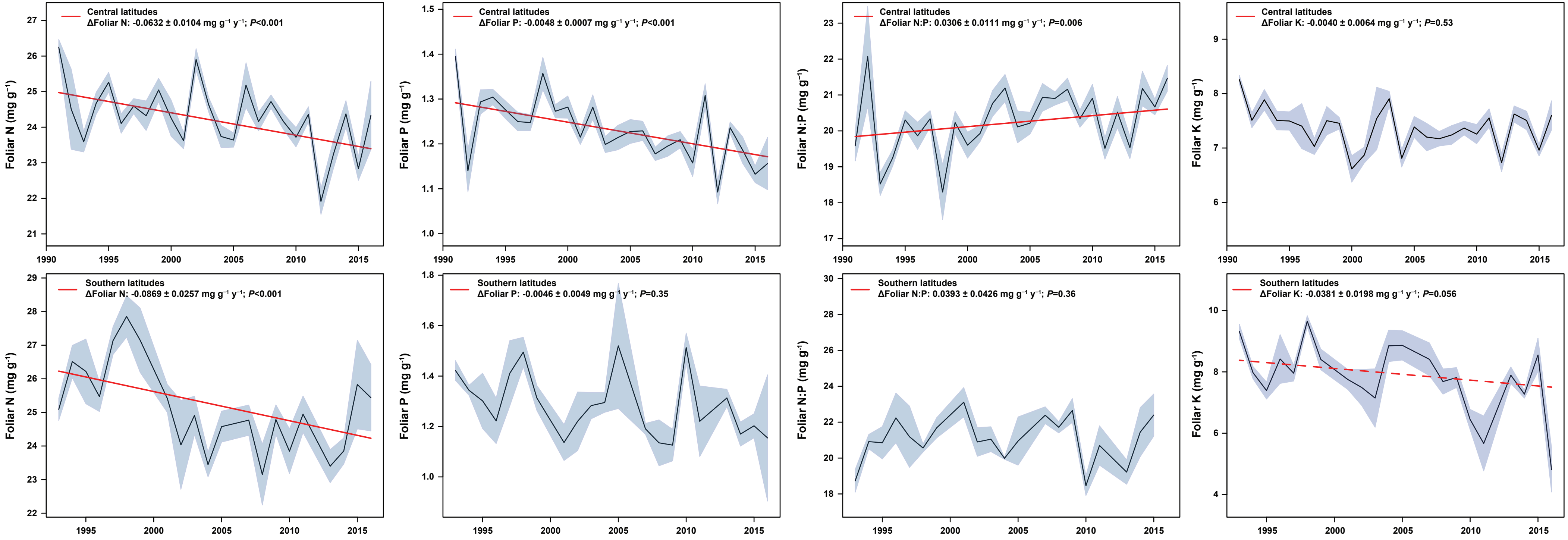

Central latitudes
ΔFoliar N: -0.0632 ± 0.0104 mg g^{-1} y^{-1}; *P*<0.001
Foliar N (mg g^{-1})
Central latitudes
ΔFoliar P: -0.0048 ± 0.0007 mg g^{-1} y^{-1}; *P*<0.001
Foliar P (mg g^{-1})
Central latitudes
ΔFoliar N:P: 0.0306 ± 0.0111 mg g^{-1} y^{-1}; *P*=0.006
Foliar N:P (mg g^{-1})
Central latitudes
ΔFoliar K: -0.0040 ± 0.0064 mg g^{-1} y^{-1}; *P*=0.53
Foliar K (mg g^{-1})
Southern latitudes
ΔFoliar N: -0.0869 ± 0.0257 mg g^{-1} y^{-1}; *P*<0.001
Foliar N (mg g^{-1})
Southern latitudes
ΔFoliar P: -0.0046 ± 0.0049 mg g^{-1} y^{-1}; *P*=0.35
Foliar P (mg g^{-1})
Southern latitudes
ΔFoliar N:P: 0.0393 ± 0.0426 mg g^{-1} y^{-1}; *P*=0.36
Foliar N:P (mg g^{-1})
Southern latitudes
ΔFoliar K: -0.0381 ± 0.0198 mg g^{-1} y^{-1}; *P*=0.056
Foliar K (mg g^{-1})

Supplementary Figure 10. **Shifts of the elementome/bioelemental composition of European tree species toward lower nutrient concentrations in recent decades in the PCA** of the foliar elemental concentrations and N:P ratio for the six dominant species, *Pinus sylvestris*, *Picea abies*, *Fagus sylvatica*, *Quercus robur*, *Quercus petraea* and *Quercus ilex*, for Europe. All plots compare the data for 1990-2004withthe data for 2005-2016. The circles/ellipses for each species and period depict the mean position and the space occupied by the 95% confidence interval for the scores of each species.

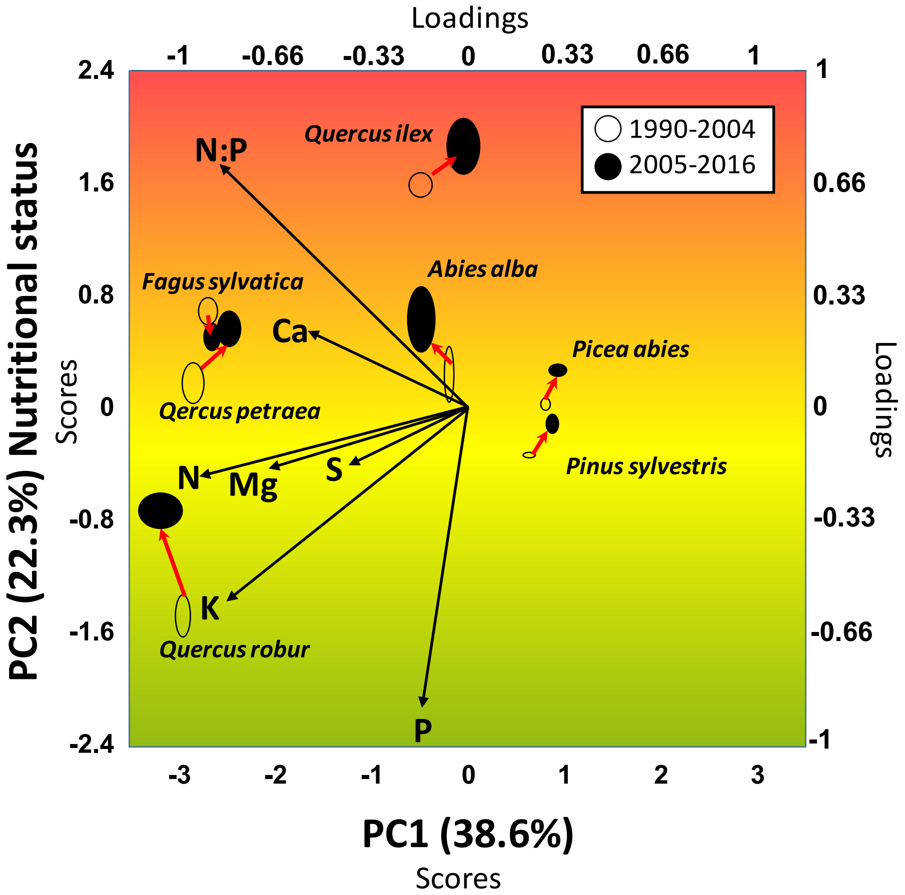

Supplementary Figure 11. **Temporal evolution and trends in N and S deposition, mean annual temperature (MAT), and mean annual precipitation (MAP).** Trends were calculated using GLMMs with random slopes, with forest as a random effect and year as a fixed effect. Models also used an ARMA (1, 0) auto correlational structure. The shaded areas indicate the 95% confidence intervals of the means (calculated as 1.96 times the standard error of the mean). See Methods for further details.

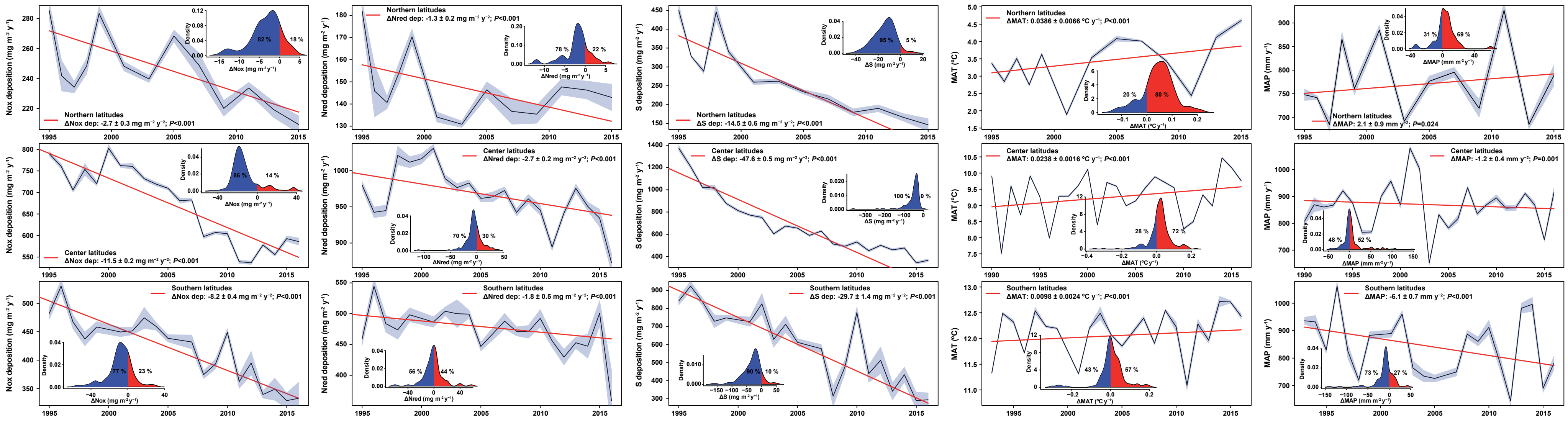

Northern latitudes
ΔNox dep: -2.7 ± 0.3 mg m⁻² y⁻²; P<0.001
82 %
18 %
Northern latitudes
ΔNred dep: -1.3 ± 0.2 mg m⁻² y⁻²; P<0.001
78 %
22 %
Northern latitudes
ΔS dep: -14.5 ± 0.6 mg m⁻² y⁻²; P<0.001
95 %
5 %
Northern latitudes
ΔMAT: 0.0386 ± 0.0066 °C y⁻¹; P<0.001
20 %
80 %
Northern latitudes
ΔMAP: 2.1 ± 0.9 mm y⁻²; P=0.024
31 %
69 %
Center latitudes
ΔNox dep: -11.5 ± 0.2 mg m⁻² y⁻²; P<0.001
86 %
14 %
Center latitudes
ΔNred dep: -2.7 ± 0.2 mg m⁻² y⁻²; P<0.001
70 %
30 %
Center latitudes
ΔS dep: -47.6 ± 0.5 mg m⁻² y⁻²; P<0.001
100 %
0 %
Center latitudes
ΔMAT: 0.0238 ± 0.0016 °C y⁻¹; P<0.001
28 %
72 %
Center latitudes
ΔMAP: -1.2 ± 0.4 mm y⁻²; P=0.001
48 %
52 %
Southern latitudes
ΔNox dep: -8.2 ± 0.4 mg m⁻² y⁻²; P<0.001
77 %
23 %
Southern latitudes
ΔNred dep: -1.8 ± 0.5 mg m⁻² y⁻²; P<0.001
56 %
44 %
Southern latitudes
ΔS dep: -29.7 ± 1.4 mg m⁻² y⁻²; P<0.001
90 %
10 %
Southern latitudes
ΔMAT: 0.0098 ± 0.0024 °C y⁻¹; P<0.001
43 %
57 %
Southern latitudes
ΔMAP: -6.1 ± 0.7 mm y⁻²; P<0.001
73 %
27 %
Nox deposition (mg m⁻² y⁻¹)
Nred deposition (mg m⁻² y⁻¹)
S deposition (mg m⁻² y⁻¹)
MAT (°C)
MAP (mm y⁻¹)
Density
ΔNox (mg m⁻² y⁻¹)
ΔNred (mg m⁻² y⁻¹)
ΔS (mg m⁻² y⁻¹)
ΔMAT (°C y⁻¹)
ΔMAP (mm m⁻² y⁻¹)

Supplementary Figure 12. **Map of the sampling sites (a) and map depicting the seven tree species** (*Fagus sylvatica, Picea abies, Abies alba, Pinus sylvestris, Quercus ilex, Quercus petrea, Quercus robur*) with highest number of observations (b). The dotted lines separate northern, central, and southern Europe in this study.

a

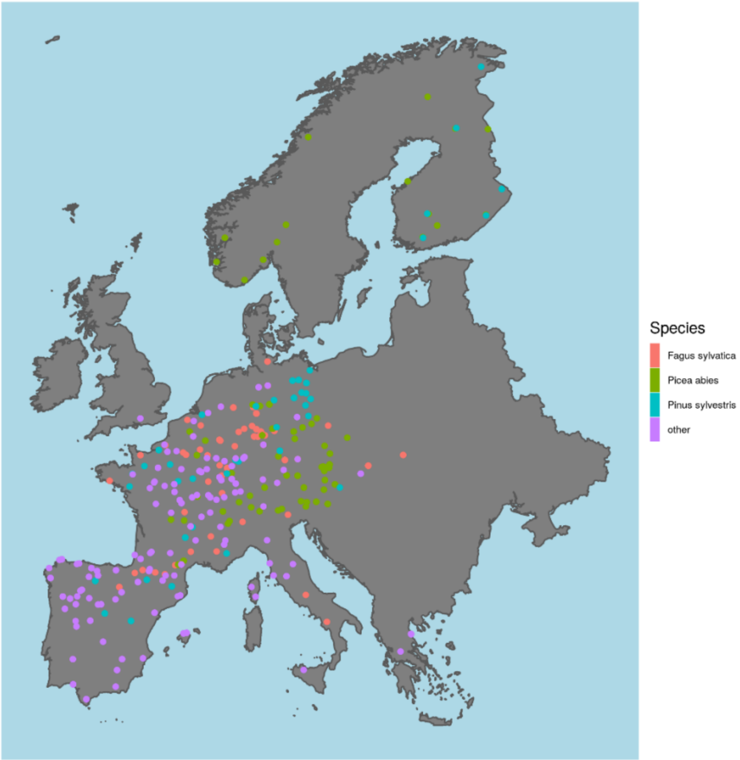

b

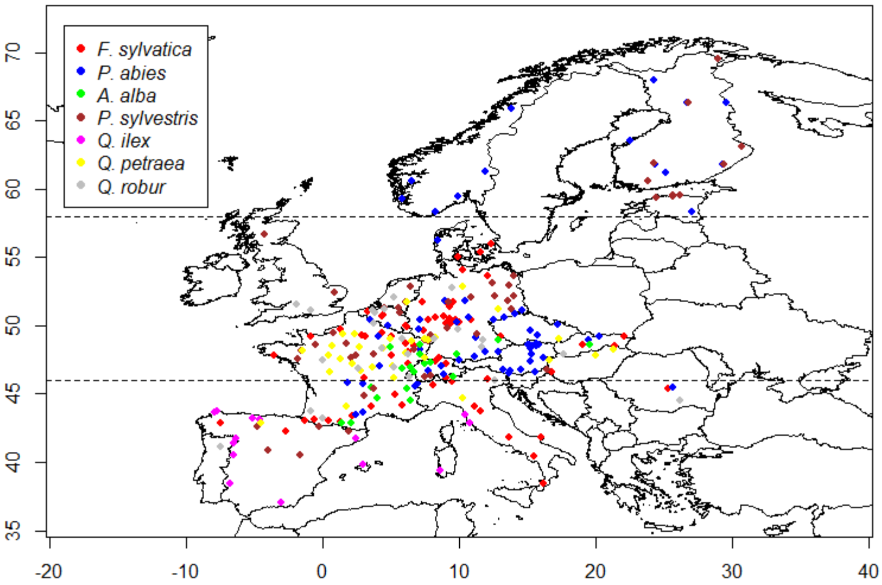

Supplementary Table 1. Summary of the models used to estimate the temporal contributions in the trends of foliar N, P and K concentrations and the N:P ratio.

Nitrogen

	Beta	SE	DF	*t*	*P*
**(Intercept)**	0.000	0.000	4151	-2.474	0.0134
**cdioxide**	0.127	0.027	4151	4.678	0.0000
**map.an**	0.021	0.004	4151	4.621	0.0000
**map.mean**	-0.048	0.095	515	-0.509	0.6110
**mat.an**	-0.011	0.014	4151	-0.830	0.4064
**mat.mean**	0.622	0.223	515	2.791	0.0054
**nox.mean**	1.890	0.289	515	6.532	0.0000
**nred.mean**	-0.082	0.207	515	-0.396	0.6924
**sdep.mean**	-0.197	0.299	515	-0.660	0.5099
**cdioxide:mat.mean**	-0.796	0.218	4151	-3.656	0.0003
**mat.an:mat.mean**	0.040	0.013	4151	2.994	0.0028
**cdioxide:nox.mean**	-1.734	0.253	4151	-6.855	0.0000
**cdioxide:sdep.mean**	0.745	0.253	4151	2.946	0.0032
**nox.mean:nred.mean**	-0.942	0.162	515	-5.830	0.0000
**map.mean:nox.mean**	0.491	0.168	515	2.921	0.0036
**mat.mean:nred.mean**	0.942	0.165	515	5.718	0.0000
**map.mean:sdep.mean**	-0.510	0.175	515	-2.911	0.0038

Number of observations: 4683
Number of groups: 525

R^2m	R^2c	phi
0.2498573	0.8947253	0.457756

Phosphorus

	Beta	SE	DF	*t*	*P*
**(Intercept)**	0.000	0.000	4161	13.48883	0
**cdioxide**	-0.080	0.014	4161	-5.62331	0
**mat.mean**	-0.206	0.028	523	-7.33994	0

Number of observations: 4687
Number of groups: 525

R^2m	R^2c	phi
0.052	0.433	0.491

N:P

	**Beta**	**SE**	**DF**	***t***	***P***
**(Intercept)**	0.000	0.000	4152	-2.254	0.024
**cdioxide**	0.099	0.022	4152	4.542	0.000
**map.mean**	-0.031	0.088	515	-0.349	0.727
**mat.an**	0.027	0.005	4152	4.968	0.000
**mat.mean**	-0.036	0.054	515	-0.656	0.512
**nox.mean**	0.551	0.270	515	2.039	0.042
**nred.mean**	-0.882	0.172	515	-5.141	0.000
**sdep.mean**	0.798	0.169	515	4.710	0.000
**cdioxide:nox.mean**	-0.863	0.233	4152	-3.707	0.000
**map.mean:nox.mean**	0.727	0.161	515	4.519	0.000
**nred.mean:sdep.mean**	-0.516	0.097	515	-5.322	0.000
**mat.mean:nred.mean**	1.341	0.165	515	8.139	0.000
**map.mean:sdep.mean**	-0.606	0.170	515	-3.576	0.000

**Number of observations: 4680**			
**Number of groups: 525**			
	***R^2*m**	***R^2*c**	**phi**
	0.257369	0.856251	0.343654

Potassium

	**Beta**	**SE**	**DF**	***t***	***P***
**(Intercept)**	0.0000	0.0000	4156	9.077	0
**cdioxide**	-0.1591	0.0263	4156	-6.039	0
**map.mean**	0.1426	0.0388	520	3.681	0.0003
**mat.an**	0.0831	0.0254	4156	3.273	0.0011
**mat.mean**	-0.0158	0.0503	520	-0.315	0.7532
**nox.an**	0.0202	0.0164	4156	1.229	0.2193
**nred.an**	0.0046	0.0115	4156	0.398	0.6907
**nred.mean**	-2.0020	0.4244	520	-4.717	0
**sdep.an**	-0.0323	0.0175	4156	-1.842	0.0655
**mat.an:mat.mean**	-0.0886	0.0251	4156	-3.531	0.0004
**cdioxide:nred.mean**	1.5401	0.3990	4156	3.860	0.0001
**nox.an:nred.an**	-0.0634	0.0157	4156	-4.028	0.0001
**nred.an:sdep.an**	0.0493	0.0158	4156	3.120	0.0018
**mat.mean:nred.mean**	0.6007	0.1531	520	3.923	0.0001

**Number of observations: 4690**	***R^2*m**	***R^2*c**	**phi**
**Number of groups: 525**	0.050	0.654	0.358

Calcium

	Beta	SE	DF	*t*	*P*
**(Intercept)**	0.0000	0.0000	4159	2.5351	0.0113
**map.an**	0.0203	0.0064	4159	3.1669	0.0016
**map.mean**	-0.0475	0.0894	517	-0.5307	0.5959
**mat.an**	0.0314	0.0060	4159	5.2135	<0.0001
**nox.an**	-0.0108	0.0087	4159	-1.2423	0.2142
**nox.mean**	0.4656	0.0863	517	5.3974	<0.0001
**nred.an**	-0.0078	0.0082	4159	-0.9582	0.3380
**nred.mean**	-0.8307	0.1529	517	-5.4337	<0.0001
**sdep.mean**	1.2530	0.1871	517	6.6960	<0.0001
**nox.an:nred.an**	0.0253	0.0082	4159	3.0982	0.0020
**map.an:nox.an**	-0.0289	0.0065	4159	-4.4589	<0.0001
**nox.mean:sdep.mean**	-0.8579	0.1425	517	-6.0216	<0.0001
**map.mean:nred.mean**	0.8084	0.1633	517	4.9506	<0.0001
**map.mean:sdep.mean**	-0.6350	0.1651	517	-3.8464	0.0001

**Number of observations: 4690**	**R^2m**	**R^2c**	**phi**
**Number of groups: 525**	0.10	0.82	0.42

Magnesium

	Beta	SE	DF	*t*	*P*
**(Intercept)**	0.0000	0.0000	4145	5.2374	<0.0001
**cdioxide**	-0.0514	0.0192	4145	-2.6820	0.0073
**map.an**	0.0353	0.0095	4145	3.7084	0.0002
**map.mean**	-0.0728	0.0861	517	-0.8453	0.3983
**mat.an**	1.2591	0.4235	4145	2.9734	0.0030
**mat.mean**	0.2477	0.0347	517	7.1370	<0.0001
**nox.an**	-0.0196	0.0169	4145	-1.1586	0.2467
**nox.mean**	-0.7069	0.1260	517	-5.6088	<0.0001
**nred.an**	-0.0063	0.0122	4145	-0.5163	0.6057
**sdep.an**	-0.0006	0.0176	4145	-0.0348	0.9723
**sdep.mean**	0.6041	0.1542	517	3.9169	0.0001
**cdioxide:mat.an**	-1.2735	0.4242	4145	-3.0021	0.0027
**nox.an:nred.an**	0.0602	0.0165	4145	3.6501	0.0003
**nred.an:sdep.an**	-0.0634	0.0164	4145	-3.8747	0.0001
**mat.an:sdep.an**	-0.1391	0.0159	4145	-8.77527	<0.0001
**map.mean:nox.mean**	0.6613	0.1522	517	4.34576	<0.0001
**map.mean:sdep.mean**	-0.6116	0.1668	517	-3.66622	0.0003
**mat.an:nox.an**	0.0424	0.0139	4145	3.046694	0.0023

**Number of observations: 4680**	**R^2m**	**R^2c**	**phi**
**Number of groups: 524**	0.10	0.62	0.28

Sulphur

	**Beta**	**SE**	**DF**	***t***	***P***
**(Intercept)**	0.0000	0.0000	4097	-2.9092	0.0036
**cdioxide**	0.1932	0.0487	4097	3.9650	0.0001
**map.mean**	0.0263	0.0751	514	0.3496	0.7267
**mat.mean**	0.0053	0.0619	514	0.0856	0.9318
**nox.mean**	1.4939	0.5608	514	2.6638	0.0080
**nred.mean**	0.2685	0.0870	514	3.0876	0.0021
**sdep.mean**	1.4732	0.2048	514	7.1942	0.0000
**cdioxide:nox.mean**	-2.5060	0.5230	4097	-4.7915	0.0000
**map.mean:nox.mean**	0.4596	0.1394	514	3.2960	0.0010
**mat.mean:nox.mean**	0.6923	0.1534	514	4.5148	0.0000
**nred.mean:sdep.mean**	-0.3340	0.0861	514	-3.8793	0.0001
**map.mean:sdep.mean**	-0.5374	0.1466	514	-3.6657	0.0003
**mat.mean:sdep.mean**	-0.7124	0.1416	514	-5.0308	0.0000

**Number of observations: 4624**	R^2m	R^2c	**phi**
**Number of groups: 525**	0.12	0.42	0.60

Supplementary Table 2. Best model accounting for the foliar nutrient concentrations at the tree level for Europe and the entire set of tree species. Note that the increase in DBH, a proxy of tree growth, was never selected, neither it was in any of the regional analyses conducted for northern, central or southern Europe.

	Intrc	CO2	MAT	Nox	Nred	Sox	CO2:MAT	CO2:Nred	CO2:Sox	MAT:Nox	MAT:Nred	MAT:Sox	Nox:Nred	Nox:Sox	Nred:Sox	r.sq1	r.sq2	df	delta	w
n	0.032	0.007	0.077	NA	0.082	0.170	-0.047	NA	0.076	NA	NA	NA	NA	NA	0.039	0.038	0.765	11	0	1
p	-0.067	-0.076	0.080	0.095	-0.039	NA	0.078	NA	NA	0.129	NA	NA	0.061	NA	NA	0.024	0.240	11	0	1
k	0.288	-0.187	-0.026	-0.090	-0.004	NA	NA	0.050	NA	0.097	-0.132	NA	NA	NA	NA	0.030	0.617	11	0	1
s	0.156	-0.035	0.000	-0.070	NA	0.187	NA	NA	0.082	NA	NA	-0.056	NA	0.069	NA	0.019	0.167	11	0	1
np	0.320	0.071	NA	-0.100	0.169	0.079	NA	-0.079	0.045	NA	NA	NA	-0.153	NA	NA	0.043	0.617	11	0	1
ca	NA	NA	NA	NA	NA	NA	NA	NA	NA	NA	NA	NA	NA	NA	NA	NA	NA	NA	NA	NA
mg	NA	NA	NA	NA	NA	NA	NA	NA	NA	NA	NA	NA	NA	NA	NA	NA	NA	NA	NA	NA

Supplementary Table 3. **Squared Mahalanobis distances (M) among the elementomes** of the species sampled in 1990-2005 and 2005-2016 in Europe, with foliar N, P, S, Ca, Mg and K concentrations and N:P ratios as variables (Fig. 5).

	Fagus sylvatica 2005-2016	*Quercus ilex* 1990-2004	*Quercus ilex* 2005-2016	*Quercus petraea* 1990-2004	*Quercus petraea* 2005-2016	*Quercus robur* 1990-2004	*Quercus robur* 2005-2016	*Abies alba* 1990-2004	*Abies alba* 2005-2016	*Picea abies* 1990-2004	*Picea abies* 2005-2016	*Pinus sylvestris* 1990-2004	*Pinus sylvestris* 2005-2016
Fagus sylvatica 1990-2004	M=0.368<br>*F*=18.4<br>*P*<0.0001	M=18.4<br>*F*=249<br>*P*<0.0001	M=22.0<br>*F*=277<br>*P*<0.0001	M=1.22 *F*=30.1<br>*P*<0.0001	M=1.39 *F*=37.7<br>*P*<0.0001	M=3.09<br>*F*=73.3<br>*P*<0.0001	M=3.98 *F*=151<br>*P*<0.0001	M=23.4<br>*F*=427<br>*P*<0.0001	M=22.8<br>*F*=560.9991<br>*P*<0.0001	M=29.1<br>*F*=1732<br>*P*<0.0001	M=29.8<br>*F*=2008.758<br>*P*<0.0001	M=25.1 *F*=1404<br>*P*<0.0001	M=22.0 *F*=1543<br>*P*<0.0001
Fagus sylvatica 2005-2016		M=16.1<br>*F*=234<br>*P*<0.0001	M=19.7<br>*F*=266<br>*P*<0.0001	M=1.79 *F*=50.7<br>*P*<0.0001	M=1.77 *F*=56.0<br>*P*<0.0001	M=3.86 *F*=104<br>*P*<0.0001	M=3.78 *F*=178<br>*P*<0.0001	M=19.7<br>*F*=396<br>*P*<0.0001	M=19.2<br>*F*=541<br>*P*<0.0001	M=25.8<br>*F*=2218<br>*P*<0.0001	M=26.5<br>*F*=2752<br>*P*<0.0001	M=22.9 *F*=1799<br>*P*<0.0001	M=19.8 *F*=2178<br>*P*<0.0001
Quercus ilex 1990-2004			M=1.09<br>*F*=8.49<br>*P*<0.0001	M=20.2 *F*=227<br>*P*<0.0001	M=16.2 *F*=188<br>*P*<0.0001	M=25.1 *F*=275<br>*P*<0.0001	M=24.2 *F*=322<br>*P*<0.0001	M=3.19<br>*F*=30.8<br>*P*<0.0001	M=1.89<br>*F*=21.1<br>*P*<0.0001	M=5.33<br>*F*=81.2<br>*P*<0.0001	M=5.45<br>*F*=85.6<br>*P*<0.0001	M=7.57 *F*=113<br>*P*<0.0001	M=8.16 *F*=129<br>*P*<0.0001
Quercus ilex 2005-2016				M=25.3 *F*=268<br>*P*<0.0001	M=19.5 *F*=214<br>*P*<0.0001	M=30.7 *F*=319<br>*P*<0.0001	M=29.6*F*=368<br>*P*<0.0001	M=4.18<br>*F*=38.3<br>*P*<0.0001	M=3.23<br>*F*=34.1<br>*P*<0.0001	M=4.72<br>*F*=66.5<br>*P*<0.0001	M=4.34<br>*F*=63.0<br>*P*<0.0001	M=5.94 *F*=82.4<br>*P*<0.0001	M=6.99 *F*=102<br>*P*<0.0001
Quercus petraea 1990-2004					M=1.03 *F*=19.7<br>*P*<0.0001	M=1.21<br>*F*=21.1<br>*P*<0.0001	M=2.67<br>*F*=64.241<br>*P*<0.0001	M=26.1<br>*F*=374<br>*P*<0.0001	M=25.3<br>*F*=453<br>*P*<0.0001	M=31.1<br>*F*=972<br>*P*<0.0001	M=32.3<br>*F*=1076<br>*P*<0.0001	M=27.7*F*=838<br>*P*<0.0001	M=24.5 *F*=832<br>*P*<0.0001
Quercus petraea 2005-2016						M=3.30<br>*F*=61.5<br>*P*<0.0001	M=4.29 *F*=113<br>*P*<0.0001	M=22.8<br>*F*=344<br>*P*<0.0001	M=21.6<br>*F*=414<br>*P*<0.0001	M=26.5<br>*F*=934<br>*P*<0.0001	M=27.3<br>*F*=1036<br>*P*<0.0001	M=23.1 *F*=785<br>*P*<0.0001	M=20.5 *F*=796<br>*P*<0.0001
Quercus robur 1990-2004							M=1.53<br>*F*=35.3<br>*P*<0.0001	M=29.7<br>*F*=415<br>*P*<0.0001	M=2829<br>*F*=504<br>*P*<0.0001	M=35.4<br>*F*=1048<br>*P*<0.0001	M=36.9<br>*F*=1162<br>*P*<0.0001	M=31.3 *F*=898<br>*P*<0.0001	M=27.7 *F*=888<br>*P*<0.0001
Quercus robur 2005-2016								M=25.7<br>*F*=459<br>*P*<0.0001	M=25.6<br>*F*=613<br>*P*<0.0001	M=31.5<br>*F*=1745<br>*P*<0.0001	M=33.1<br>*F*=2066<br>*P*<0.0001	M=28.0 *F*=1462<br>*P*<0.0001	M=24.2 *F*=1563<br>*P*<0.0001
Abies alba 1990-2004									M=0.579<br>*F*=8.24<br>*P*<0.0001	M=2.47<br>*F*=53.1<br>*P*<0.0001	M=2.79<br>*F*=62.8<br>*P*<0.0001	M=5.20*F*=109<br>*P*<0.0001	M=5.21 *F*=119<br>*P*<0.0001
Abies alba 2005-2016										M=4.16<br>*F*=129<br>*P*<0.0001	M=4.55<br>*F*=150<br>*P*<0.0001	M=7.59 *F*=228<br>*P*<0.0001	M=7.87 *F*=265<br>*P*<0.0001
Picea abies 1990-2004											M=0.0917<br>*F*=14.3<br>*P*<0.0001	M=1.41 *F*=148<br>*P*<0.0001	M=2.01 *F*=343<br>*P*<0.0001

Picea abies 2005-2016		M=1.25 *F*=166 *P*<0.0001	M=1.97 *F*=510 *P*<0.0001
Pinussylvestris 1990-2004			M=0.267 *F*=38.4 *P*<0.0001

Supplementary Table 4. **Main effects of the variables in the DA for the entire Europe** (Fig. 5). Wilks' λ and *P* value of the discriminant functional analysis among the species sampled in 1990-2005 and 2005-2016 in Europe, with foliar N, P, S, Ca, Mg and K concentrations and N:P ratios as variables.

Variable	Wilks' λ	*F*	*P*
[N]	0.700	454	<0.0001
[S]	0.992	9.09	<0.0001
[P]	0.967	36.2	<0.0001
[Ca]	0.888	134	<0.0001
[Mg]	0.838	205	<0.0001
[K]	0.890	131	<0.0001
[N]:[P]	0.965	39.0	<0.0001

Supplementary Table 5. **Squared Mahalanobis distances (M) among the elementomes of the species sampled in 1990-2005 and 2005-2016 in southern Europe**, with foliar N, P, S, Ca, Mg and K concentrations and N:P ratios as variables (Fig. 5).

	Fagus sylvatica 2005-2016	*Quercus ilex* 1990-2004	*Quercus ilex* 2005-2016	*Abies alba* 1990-2004	*Abies alba* 2005-2016	*Picea abies* 1990-2004	*Picea abies* 2005-2016	*Pinus sylvestris* 1990-2004	*Pinus sylvestris* 2005-2016
Fagus sylvatica 1990-2004	M=2.19 *F*=18.8652 *P*<0.0001	M=27.2 *F*=202 *P*<0.0001	M=28.8 *F*=206 *P*<0.0001	M=32.4 *F*=174 *P*<0.0001	M=31.2 *F*=213 *P*<0.0001	M=32.5 *F*=173 *P*<0.0001	M=42.3 *F*=280 *P*<0.0001	M=33.3 *F*=156 *P*<0.0001	M=34.3 *F*=173 *P*<0.0001
Fagus sylvatica 2005-2016		M=18.6 *F*=176 *P*<0.0001	M=18.7 *F*=169 *P*<0.0001	M=24.6 *F*=157 *P*<0.0001	M=22.8 *F*=193 *P*<0.0001	M=25.0 *F*=157 *P*<0.0001	M=32.8 *F*=269 *P*<0.0001	M=26.0 *F*=141 *P*<0.0001	M=25.9 *F*=153 *P*<0.0001
Quercus ilex 1990-2004			M=1.09 *F*=8.45 *P*<0.0001	M=1.99 *F*=11.4 *P*<0.0001	M=1.14 *F*=8.41 *P*<0.0001	M=4.02 *F*=22.7 *P*<0.0001	M=4.70 *F*=33.5 *P*<0.0001	M=3.17 *F*=15.6 *P*<0.0001	M=2.06 *F*=11.0 *P*<0.0001
Quercus ilex 2005-2016				M=3.95 *F*=21.8 *P*<0.0001	M=2.76 *F*=19.5 *P*<0.0001	M=6.09 *F*=33.3 *P*<0.0001	M=5.57 *F*=38.3 *P*<0.0001	M=5.21 *F*=25.0 *P*<0.0001	M=2.86 *F*=14.8 *P*<0.0001
Abies alba 1990-2004					M=0.651 *F*=3.47 *P*=0.001	M=2.31 *F*=10.1 *P*<0.0001	M=2.35 *F*=12.3 *P*<0.0001	M=3.10 *F*=12.2 *P*<0.0001	M=2.50 *F*=10.4 *P*<0.0001
Abies alba 2005-2016						M=4.56 *F*=24.1 *P*<0.0001	M=4.50 *F*=29.5 *P*<0.0001	M=4.43 *F*=20.6 *P*<0.0001	M=3.29 *F*=16.4 *P*<0.0001
Piceaabies 1990-2004							M=1.19 *F*=6.14 *P*<0.0001	M=1.43 *F*=5.58 *P*<0.0001	M=2.01 *F*=8.33 *P*<0.0001
Piceaabies 2005-2016								M=2.33 *F*=10.6 *P*<0.0001	M=1.74 *F*=8.33 *P*<0.0001
*Pinussylvestris*1990-2004									M=0.607 *F*=2.28 *P*=0.027

Supplementary Table 6**. Main effects of the variables in the DA**. Wilks' λ and *P* value for the discriminant functional analysis among the species sampled in 1990-2005 and 2005-2014 in southern Europe, with foliar N, P, S, Ca, Mg and K concentrations and N:P ratios as variables (Fig. 5).

Variable	Wilks' λ	*F*	*P*
[N]	0.646	53.3	<0.0001
[S]	0.815	22.1	<0.0001
[P]	0.908	9.89	<0.0001
[Ca]	0.854	16.6	<0.0001
[Mg]	0.927	7.71	<0.0001
[K]	0.942	5.95	<0.0001
[N]:[P]	0.883	12.8	<0.0001

Supplementary Table 7. **Squared Mahalanobis distances (M) among the elementomes of the species sampled in 1990-2005 and 2005-2014 in central Europe**, with foliar N, P, S, Ca, Mg and K concentrations and N:P ratios as variables (Fig. 5).

	Fagus sylvatica 2005-2016	*Quercus petraea* 1990-2004	*Quercus petraea* 2005-2016	*Quercus robur* 1990-2004	*Quercus robur* 2005-2016	*Abies alba* 1990-2004	*Abies alba* 2005-2016	*Picea abies* 1990-2004	*Picea abies* 2005-2016	*Pinus sylvestris* 1990-2004	*Pinus sylvestris* 2005-2016
Fagus sylvatica 1990-2004	M=0.610 *F*=25.0 *P*<0.0001	M=1.44 *F*=31.6 *P*<0.0001	M=1.21 *F*=29.2 *P*<0.0001	M=3.59 *F*=74.0 *P*<0.0001	M=4.61 *F*=151 *P*<0.0001	M=23.4 *F*=283 *P*<0.0001	M=23.4 *F*=387 *P*<0.0001	M=28.1 *F*=1329 *P*<0.0001	M=29.7 *F*=1647 *P*<0.0001	M=22.7 *F*=1013 *P*<0.0001	M=21.4 *F*=1246.116 *P*<0.0001
Fagus sylvatica 2005-2016		M=1.95 *F*=49.8 *P*<0.0001	M=2.17 *F*=62.2 *P*<0.0001	M=4.01 *F*=95.2 *P*<0.0001	M=3.90 *F*=161 *P*<0.0001	M=20.6 *F*=270 *P*<0.0001	M=20.8 *F*=384 *P*<0.0001	M=26.3 *F*=1776 *P*<0.0001	M=28.0 *F*=2403 *P*<0.0001	M=21.8 *F*=1362 *P*<0.0001	M=20.2 *F*=1869.772 *P*<0.0001
Quercus petraea 1990-2004			M=1.28 *F*=22.9 *P*<0.0001	M=1.24 *F*=19.5 *P*<0.0001	M=2.93 *F*=64.4 *P*<0.0001	M=28.2 *F*=289.5 *P*<0.0001	M=28.0 *F*=372 *P*<0.0001	M=32.6 *F*=906 *P*<0.0001	M=34.6 *F*=1053 *P*<0.0001	M=27.8 *F*=746 *P*<0.0001	M=25.8 *F*=807.267 *P*<0.0001
Quercus petraea 2005-2016				M=3.57 *F*=60.4 *P*<0.0001	M=4.62 *F*=112 *P*<0.0001	M=23.8 *F*=255 *P*<0.0001	M=23.1 *F*=324 *P*<0.0001	M=27.1 *F*=854 *P*<0.0001	M=28.7 *F*=1003 *P*<0.0001	M=22.7 *F*=687 *P*<0.0001	M=21.3 *F*=768.741 *P*<0.0001
Quercus robur 1990-2004					M=1.61 *F*=33.3 *P*<0.0001	M=32.3 *F*=321 *P*<0.0001	M=32.0 *F*=409 *P*<0.0001	M=37.5 *F*=962 *P*<0.0001	M=40.0 *F*=1115 *P*<0.0001	M=31.8 *F*=791 *P*<0.0001	M=29.6 *F*=846.422 *P*<0.0001
Quercus robur 2005-2016						M=28.1 *F*=341 *P*<0.0001	M=28.1 *F*=464 *P*<0.0001	M=33.9 *F*=1610 *P*<0.0001	M=36.6 *F*=2044 *P*<0.0001	M=28.6 *F*=1281 *P*<0.0001	M=26.2 *F*=1536.369 *P*<0.0001
Abies alba 1990-2004							M=0.445 *F*=3.95 *P*<0.0001	M=2.33 *F*=31.9 *P*<0.0001	M=2.87 *F*=41.0 *P*<0.0001	M=5.10 *F*=68.6 *P*<0.0001	M=4.88 *F*=70.581 *P*<0.0001
Abies alba 2005-2016								M=3.68 *F*=72.2 *P*<0.0001	M=4.28 *F*=89.4 *P*<0.0001	M=7.26 *F*=139 *P*<0.0001	M=7.14 *F*=152.071 *P*<0.0001
Picea abies 1990-2004									M=0.0948 *F*=11.2 *P*<0.0001	M=1.78 *F*=138 *P*<0.0001	M=1.86 *F*=245.150 *P*<0.0001

Picea abies<br>2005-2016		M=1.95<br>$F=201$<br>$P<0.0001$	M=2.21<br>$F=497.209$<br>$P<0.0001$
Pinus sylvestris<br>1990-2004			M=0.155<br>$F=17.537$<br>$P<0.0001$

Supplementary Table 8. **Main effects of the variables in the DA**. Wilks' λ and *P*value of the discriminant functional analysis among the species sampled in 1990-2005 and 2005-2016 in central Europe, with foliar N, P, S, Ca, Mg and K concentrations and N:P ratios as variables (Fig. 5).

Variable	Wilks' λ	*F*	*P*
[N]	0.699	446	<0.0001
[S]	0.991	8.93	<0.0001
[P]	0.958	46.0	<0.0001
[Ca]	0.902	113	<0.0001
[Mg]	0.841	196	<0.0001
[K]	0.875	149	<0.0001
[N]:[P]	0.967	35.3	<0.0001

Supplementary Table 9**. Squared Mahalanobis distances (M) among the elementomes of the species sampled in 1990-2005 and 2005-2016 in northern Europe**, with foliar N, P, S, Ca, Mg and K concentrations and N:P ratios as variables (Fig. 5).

	Picea abies 2005-2016	*Pinus sylvestris* 1990-2004	*Pinus sylvestris* 2005-2016
Picea abies 1990-2004	M=0.430<br>*F*=12.5<br>*P*<0.0001	M=5.76*F*=132<br>*P*<0.0001	M=6.68*F*=153<br>*P*<0.0001
Picea abies 2005-2016		M=6.05<br>*F*=144<br>*P*<0.0001	M=5.82*F*=139<br>*P*<0.0001
Pinus sylvestris 1990-2004			M=1.19<br>*F*=23.2<br>*P*<0.0001

Supplementary Table 10. **Main effects of the variables in the DA**. Wilks' λ and *P* value of the discriminant functional analysis among the species sampled in 1990-2004 and 2005-2016 in northern Europe, with foliar N, P, S, Ca, Mg and K concentrations and N:P ratios as variables (Fig. 5).

Variable	Wilks' λ	*F*	*P*
[N]	0.923	37.9	<0.0001
[S]	0.929	34.6	<0.0001
[P]	0.965	16.5	<0.0001
[Ca]	0.701	193	<0.0001
[Mg]	0.874	65.2	<0.0001
[K]	0.763	141	<0.0001
[N]:[P]	0.941	28.3	<0.0001

Supplementary information

1. Models to assess the temporal contributions of the environmental factors to the temporal trends of foliar nutrient concentrations at the site level

Saturated formulation of the models: foliar nutrient ~ (mean annual precipitation + precipitation annual anomaly + CO_2)^2 + (mean annual temperature + temperature annual anomaly + CO_2)^2 + (mean oxidised N deposition + oxidised N deposition annual anomaly + CO_2)^2 + (mean reduced N deposition + reduced N deposition annual anomaly + CO_2)^2 + (mean S deposition + S deposition annual anomaly + CO_2)^2 + (annual anomalies of CO_2 + annual anomalies of temperature + annual anomalies of precipitation + annual anomalies of oxidised N deposition + annual anomalies of reduced N deposition + annual anomalies of S deposition)^2 + (mean annual temperature + mean annual precipitation + mean annual oxidised N deposition + mean annual reduced N deposition + mean annual of S deposition)^2, where ^2 indicates a first-order interaction of the variables within the brackets.

2. Selection of models that best accounted for the foliar nutrient concentrations at the tree level

We selected the models conducted at the tree level that best accounted for the foliar nutrient concentrations (N, P, K, Mg, Ca and S) and the N:P ratio based on second-order Akaike information criteria (AIC). All analyses started with a saturated model containing mean annual increase in the basal area of trees (diff_basalArea), mean annual precipitation (MAP), mean annual temperature (MAT), CO_2concentration (CO_2) and deposition of nutrients (Nox.total_dep, Nred.total_dep and Sox.total_dep). The saturated model also included the second-order interactions of the predictors.

We also constructed different subset models to identify patterns at the latitudinal or species level. We used species and tree ID nested inside plot and country as random factors for the continental analysis and subsets by latitude and used only tree ID nested inside plot and country as a random factor for subsets including only one species. All analyses were performed in R ¯using the MuMIn and lme4packages.

We calculated the importance of each predictor as the sum of the AIC-based weight of the models that included the predictor.

3. Interannual variability in foliar concentration data

The trends reported in this study were found despite the strong interannual variability in foliar concentrations clearly shown in Figures, for example see figure 1. Even continuously measured sites present a relatively large interannual variability in their foliar concentrations. There are many factors contributing to these temporal variability, among which we can find: i) measurement error (we will never find two leaves with the exact same concentration of elements) + the systematic error of the sampling and analytical techniques, and ii) environmental variability can drastically affect element composition from year to year (e.g., late frosts, drought, storms or pests), iii) the inclusion of different number of sites within each year.

4. PRISMA flow diagrams of the meta-analysis for N, P and N:P

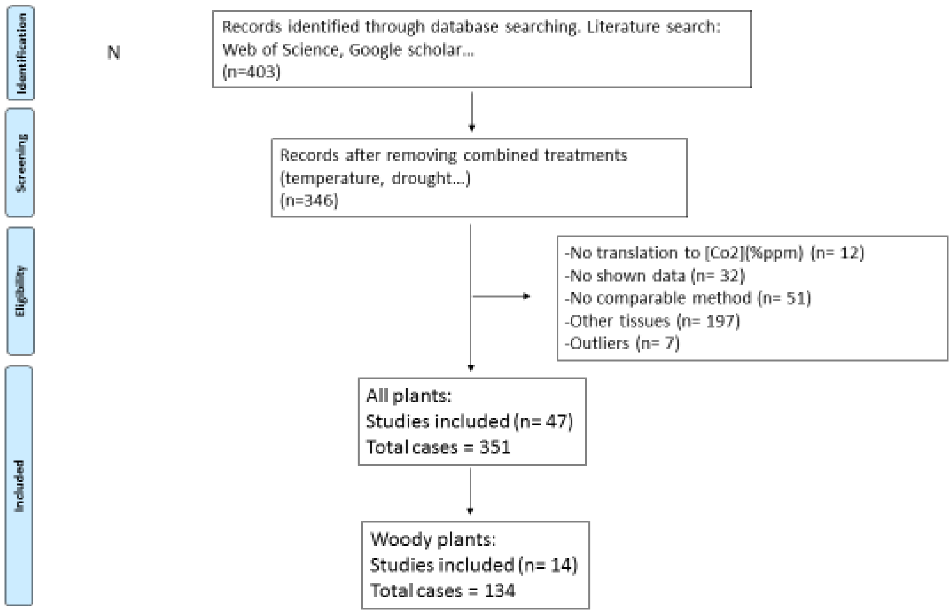

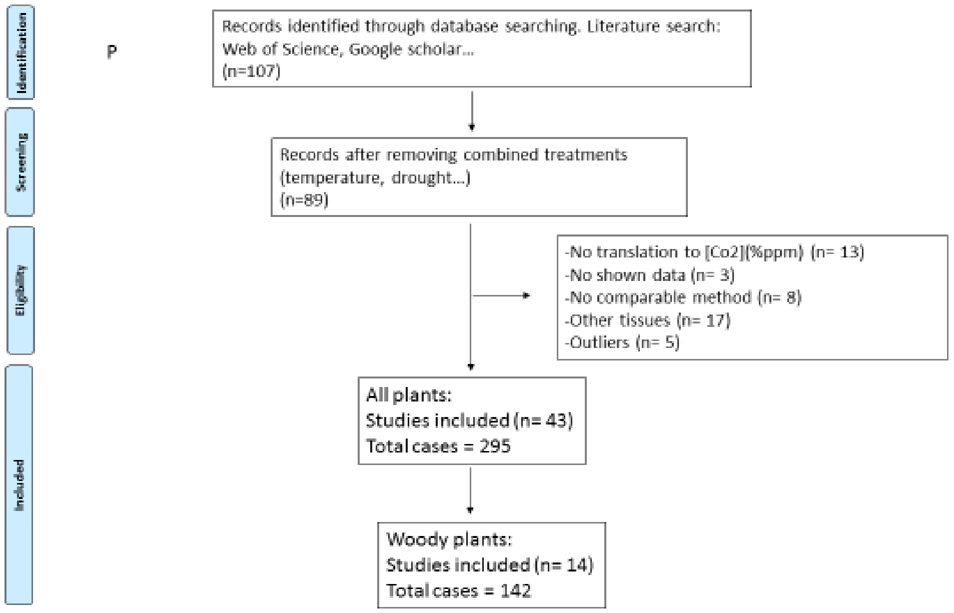

Supplementary references used for Figure 6b

1. Alberton, O., Kuyper, T. W. & Gorissen, A. Competition for nitrogen between Pinus sylvestris and ectomycorrhizal fungi generates potential for negative feedback under elevated CO2. Plant and soil. 296 (1-2), 159-172 (2007).
2. Barnes, J. D. & Pfirrmann, T. The influence of CO2 and =3, singly and in combination, on gas exchange, growth and nutrient status of radish (Raphanus sativus L.). New Phytol. 121 (3), 403-412 (1992).
3. Baslam, M.,Garmendia, I. & Goicoechea, N. Elevated CO2 may impair the beneficial effect of arbuscular mycorrhizal fungi on the mineral and phytochemical quality of lettuce. Annals of Applied Biology. 161 (2), 180-191 (2012).
4. Baxter, R., Ashenden, T. W. & Farrar, J. F. Effect of elevated CO2 and nutrient status on growth, dry matter partitioning and nutrient content of Poa alpina var vivipara L. Journal of Experimental Botany. 312, 1477-1486 (1997).
5. Blank, R. R. & Derner, J. D. Effects of CO2 enrichment on plant-soil relationships of Lepidium latifolium. Plant and soil. 262 (1-2), 159-167 (2004).
6. Blank, R. R., Morgan, T., Ziska, L. H. & White, R. H. Effect of Atmospheric CO2 levels on nutrients in cheatgrass tissue. Natural resources and environmental issues. 16, 18 (2011).
7. Brown, K. R. Carbon-dioxide enrichment accelerates the decline in nutrient status and relative growth-rate of Populus-tremuloides michx seedlings. Tree physiology. 8 (2), 161-173 (1991).
8. Cao, W. X. & Tibbitts, T. W. Starch concentration and impact on specific leaf weight and element concentrations in potato leaves under varied carbon dioxide and temperature. Journal of Plant Nutrition. 20 (7-8), 871-881 (1997).
9. Cao, B., Dang, Q. & Yue, X. Effects Of [CO2] and nitrogen on morphological and biomass traits of white birch (Betula papyrifera) seedlings. Forest Ecology and Management. 254 (2), 217-224 (2008).

10. Carlisle, E., Myers, S., Raboy, V. & Bloom, A. The effects of inorganic nitrogen form and CO2 concentration on wheat yield and nutrient accumulation and distribution. Frontiers in Plant Science. 3, 195 (2012).
11. Dijkstra, F. A. *et al.* Contrasting effects of elevated CO_2 and warming on nitrogen cycling in a semiarid grassland. *New Phytol.* **187**, 426-437 (2010).
12. Dijkstra, F. A. *et al.* Climate change alters stoichiometry of phosphorus and nitrogen in a semiarid grassland. *New Phytol.* **196**, 807-815 (2012).
13. Fangmeier, A., Gruters, U., Hogy, P., Vermehren, B. & Jager, H. J. Effects of elevated CO_2, nitrogen supply and tropospheric ozone on spring wheat .II. Nutrients (N, P, K, S, Ca, Mg, Fe, Mn, Zn). *Environ. Pollut.* **96**, 43-59 (1997).
14. Fangmeier, A., De Temmerman, L., Black, C., Persson, K. & Vorne, V. Effects of elevated CO_2 and/or ozone on nutrient concentrations and nutrient uptake of potatoes. *Eur. J. Agron.* **17**, 353-368 (2002).
15. Fleisher, D. H., Barnaby, J., Sicher, R., Resop`, J.P., Timlin, D. J. & Reddy, V. R. Effects of elevated CO2 and cyclic drought on potato under varying radiation regimes. Agricultural and Forest Meteorology. 171, 270-280 (2013)
16. Gavito, M. E., Curtis, P. S., Mikkelsen, T. N., & Jakobsen. Interactive effects of soil temperature, atmospheric carbon dioxide and soil N on root development, biomass and nutrient uptake of winter wheat during vegetative growth. Journal of Experimental Botany. 52 (362), 1913-1923 (2001).
17. Gavito, M. E. & Azcon-Aguilar, C. Temperature stress in arbuscular mycorrhizal fungi: a test for adaptation to soil temperature in three isolates of Funneliformis mosseae from different climates. Agricultural and Food Science. 21 (1), 2-11 (2012).
18. Gentile, R., Dodd, M., Lieffering, M., Brock, S. C., Theobald, P. W. & Newton P. C. D. Effects of long-term exposure to enriched CO2 on the nutrient-supplying capacity of a grassland soil. 48 (3), 357-362 (2012).
19. Gries, C., Kimball, B. A. & Idso, S.B. Nutrient-uptake during the course of a year by sour orange trees growing in ambient and elevated atmospheric carbon-dioxide concentrations. Journal of Plant Nutrition. 16 (1), 129-147 (1993).
20. Hattas, D., Stock, W. D., Mabusela, W. T. & Green I. R. Phytochemical changes in leaves of subtropical grasses and fynbos shrubs at elevated atmospheric CO2 concentrations. Global and Planetary Change. 47 (2-4), 181-192 (2005).
21. Heagle, A. S., Miller, J. E., Sherrill, D. E. & Rawlings, J. O. Effects of ozone carbon-dioxide mixtures on 2 clones of white clover. New Phytologist. 123 (4), 751-762 (1993).
22. Heagle A. S., Miller J. E., Pursley W. A. (2003). Growth and yield responses of potato to mixtures of carbon dioxide and ozone. *J. Environ. Qual.* 32 1603–1610. 10.2134/jeq2003.1603
23. Heijmans, M. M. P. D., Klees, H., de Visser, W. & Berendse, F. Response of a Sphagnum bog plant community to elevated CO2 and N supply. Plant Ecology. 162 (1), 123-134 (2002).
24. Housman, D. C., Killingbeck, K. T., Evans, R. D., Charlet, T. N. & Smith, S. D. Foliar nutrient resorption in two Mojave Desert shrubs exposed to Free-Air CO2 Enrichment (FACE). Journal of Arid Environments. 78, 26-32 (2012).
25. Huluka, G., Hileman, D. R., Biswas, P. K., Lewin, K.F., Nagy, J. & Hendrey, G. R. Effects of elevated Co2 and water-stress on mineral concentration of cotton. Agricultural and Forest Meteorology. 70 (1-4), 141-152 (1994).
26. Jifon, J. L. & Wolfe, D. W. Photosynthetic acclimation to elevated CO_2 in *Phaseolus vulgaris* L. Is altered by growth response to nitrogen supply. *Global Change Biol.* **8**, 1018-1027 (2002).
27. Jin, J., Tang, C., Armstrong, R. & Sale, P. Phosphorus supply enhances the response of legumes to elevated CO_2 (FACE) in a phosphorus-deficient vertisol. *Plant Soil* **358**, 86-99 (2012).

28. Johanson, D. W., Hungate, B. A., Dijkstra, P., Hymus, G., Hinkle, C. R., Stiling, P. & Drake, B. G. The effects of elevated CO2 on nutrient distribution in a fire-adapted scrub oak forest. 13 (5), 1388-1399 (2003).
29. Johnson, D. W., Walker, R.F. & Ball, J. T. Combined effects of nitrogen and elevated CO2 on soils from controlled environment studies. Water Air and Soil Pollution. 85 (3), 1551-1556 (1995).
30. Johanson, D. W., Hungate, B. A., Dijkstra, P., Hymus, G., Hinkle, C. R., Stiling, P. & Drake, B. G. The effects of elevated CO2 on nutrient distribution in a fire-adapted scrub oak forest. 13 (5), 1388-1399 (2003).
31. Johnson, D. W., Ball, T. & Walker, R. F. Effects of elevated Co2 and Nitrogen on Nutrient-Uptake in Ponderosa pine-seedlings. Plant and Soil. 168, 535-545 (1995).
32. Jongen, M., Fay, P. & Jones, M. B. Effects of elevated carbon dioxide and arbuscular mycorrhizal infection on Trifolium repens. New Phytologist. 132 (3), 413-423 (1996).
33. Kanowski, J. Effects of elevated CO_2 on the foliar chemistry of seedlings of two rainforest trees from north-east Australia: Implications for folivorous marsupials. *Austral. Ecol.* **26**, 165-172 (2001).
34. Kasurinen, A., Kokko-Gonzales, P., Riikonen, J., Vapaavuori, E. & Holopainen, T. Soil CO2 efflux of two silver birch clones exposed to elevated CO2 and O-3 levels during three growing seasons. Global Change Biology. 10 (10), 1654-1665 (2004).
35. Keutgen, N. & Chen, K. Responses of *Citrus* leaf photosynthesis, chlorophyll fluorescence, macronutrient and carbohydrate contents to elevated CO_2 *J. Plant Physiol.* **158**, 1307-1316 (2001).
36. Keutgen, N., Chen, K. & Lenz, F. Responses of strawberry leaf photosynthesis, chlorophyll fluorescence and macronutrient contents to elevated CO_2 *J. Plant Physiol.* **150**, 395-400 (1997).
37. Le Thiec, D., Dixon, M., Loosveldt, P & Garrec, J. P. Seasonal and annual variations of phosphorus, calcium, potassium and manganese content in different cross-sections of Picea abies (L.) Karst. Needles and Quercus rubra L leaves exposed to elevated CO2. Trees. 10 (2), 55-62 (1995).
38. Li, J., Zhou, J. M., Duan, Z. Q., Du, C. W. & Wang, H. Y. Effect of CO_2 enrichment on the growth and nutrient uptake of tomato seedlings. *Pedosphere* **17**, 343-351 (2007).
39. Lieffering, M., Kim, H. Y., Kobayashi, K. & Okada, M. The impact of elevated CO_2 on the elemental concentrations of field-grown rice grains. *Field Crops Res.* **88**, 279-286 (2004).
40. Liu, J. X., Zhang, D. Q., Zhou, G. Y. & Duan, H. L. Changes in leaf nutrient traits and photosynthesis of four tree species: effects of elevated [CO2], N fertilization and canopy positions. Journal of Plant Ecology. 5 (4), 376-390 (2012).
41. Luomala, E. M., Laitinen, K., Sutinen, S., Kellomaki, S. & Vapaavuori, E. Stomatal density, anatomy and nutrient concentrations of Scots pine needles are affected by elevated CO_2 and temperature. *Plant Cell Environ.* **28**, 733-749 (2005).
42. Manderscheid, R., Bender, R. & Jager, H. J. Effects of season long Co2 enrichment on cereals .2. nutrient concentrations and grain quality. Agriculture Ecosystems & Environment. 54 (3), 175-185 (1995).
43. McKeehen, J. D., Smart, D. J., Mackowiak, C. L., Wheeler, R. M. & Nielsen, S. S. Effect of CO2 levels on nutrient content of lettuce and radish. Natural and Artificial Ecosystems. 18 (4-5), 85-92 (1996).
44. Mjwara, J. M., Botha, C. E. J. & Radloff, S. E. Photosynthesis, growth and nutrient changes in non-nodulated Phaseolus vulgaris grown under atmospheric and elevated carbon dioxide conditions. Physiologia Plantarum. 97 (4), 754-763 (1996).
45. Morgan, J. A., Knight, W. G., Dudley, L. M. & Hunt, H. W. Enhanced root-system C-sink activity, water relations and aspects of nutrient acquisition in mycotrophic bouteloua-gracilis subjected to Co2 enrichment. Plant and Soil. 165 (1), 139-146 (1994).

46. Murray, M. B., Smith, R. I., Friend, A. & Jarvis, P. G. Effect of elevated [CO2] and varying nutrient application rates on physiology and biomass accumulation of Sitka spruce (Picea sitchensis). Tree Physiology. 20 (7), 421-434 (2000).
47. Newbery, R. M., Wolfenden, J., Mansfield, T. A. & Harrison, A. F. Nitrogen, phosphorus and potassium uptake and demand in *Agrostis capillaris*: The influence of elevated CO_2 and nutrient supply. *New Phytol.* **130**, 565-574 (1995).
48. Niklaus PA, Körner C. Synthesis of a six-year study of calcareous grasland response to in CO2 enrichment. Ecol Monographs 74, 491-511.
49. Niklaus, P. A., Leadley, P. W., Stocklin, J. & Korner, C. Nutrient relations in calcareous grassland under elevated CO_2. *Oecologia* **116**, 67-75 (1998).
50. Norisada, M., Motoshige, T., Kojima, K. & Tange, T. Effects of phosphate supply and elevated CO_2 on root acid phosphatase activity in *Pinus densiflora* seedlings. *J. Plant Nutr. Soil Sci.* **169**, 274-279 (2006).
51. Novotny, A. M., Schade, J. D., Hobbie, S. E., Kay, A. D., Kyle, M., Reich, P. B. & Elser, J. J. Stoichiometric response of nitrogen-fixing and non-fixing dicots to manipulations of CO2, nitrogen, and diversity. Oecologia. 151 (4), 687-696 (2007).
52. Nowak, J., Sroka, S. & Matysiak, B. Effects of light level, CO2 enrichment, and concentration of nutrient solution on growth, leaf nutrient content, and chlorophyll fluorescence of Boston Fern microcuttings. Journal of Plan Nutrition. 25 (10), 2161-2171 (2002).
53. Olsrud, M., Carlsson, B. A., Svensson, B., Michelsen, A. & Melillo, J. M. Responses of fungal root colonization, plant cover and leaf nutrients to long-term exposure to elevated atmospheric CO2 and warming in a subarctic birch forest understory. Global Change Biology. 16 (6), 1820-1829 (2010).
54. Owensby, C. E., Coyne, P. I., Ham, J. M., Auen, L. M. & Knapp, A. K. Biomass production in a tallgrass praire ecosystem exposed to ambient and elevated Co2. Ecological Applications. 3 (4), 644-653 (1993).
55. Pal, M. Karthikeyapandian V, Jain V, Srivastava AC, Raj A, Sengupta UK. Biomass production and nutritional levels of berseem (*Trifolium alexandrium*) grown under elevated CO_2. *Agric. Ecosyst. Environ.* **101**, 31-38 (2004).
56. Peñuelas, J., Idso, S. B., Ribas, A. & Kimball, B. A. Effects of long-term atmospheric CO2 enrichment on the mineral concentration of Citrus aurantium leaves. New Phytologist. 135 (3), 439-444 (1997).
57. Peñuelas, J., Filella, I. & Tognetti, R. Leaf mineral concentrations of Erica arborea, Juniperus communis and Myrtus communis growing in the proximity of a natural CO2 spring. Global Change Biology. 7 (3), 291-301 (2001).
58. Pfirrmann, T., Barnes, J. D., Steiner, K., Schramel, P., Busch, U., Kuchenhoff, H. & Payer, H. D. Effects of elevated CO2, O-3 and K deficiency on Norway spruce (Picea abies): Nutrient supply, content and leaching. New Phytologist. 134 (2), 267-278 (1996).
59. Pierce, S., Stirling, C. M. & Baxter, R. Pseudoviviparous reproduction of *Poa alpina* var. Vivipara L. (poaceae) during long-term exposure to elevated atmospheric CO_2. *Ann. Bot.* **91**, 613-622 (2003).
60. Porter, M. A. & Grodzinski, B. Acclimation to high CO_2 in bean *Carbonic anhydrase* and ribulose bisphosphate carboxylase. *Plant Physiol.* **74**, 413-416 (1984).
61. Prior, S.A., Rogers, H.H., Runion, G.B., Torbert, H.A., Reicosky, D.C. Carbon dioxide-enriched agroecosystems: influence of tillage on short-term soil carbon dioxide efflux. J. Env. Qual. 26, 244–252 (1997)
66. Prior, S. A., Torbert, H. A., Runion, G. B., Rogers, H. H. & Kimball, B. A. Free-air CO2 enrichment of sorghum: Soil carbon and nitrogen dynamics. Journal of Environmental Quality. 37 (3), 753-758 (2008).
67. Roberntz, P. & Linder, S. Effects of long-term CO_2 enrichment and nutrient availability in Norway spruce. II. Foliar chemistry. *Trees* **14**, 17-27 (1999).

68. Roberntz, P. & Linder, S. Effects of long-term CO2 enrichment and nutrient availability in Norway spruce. II. Foliar chemistry. Trees-structure and function. 14 (1), 17-27 (1999).
69. Rodenkirchen, H., Goettlein, A., Kozovits, A. R., Matyssek, R. & Grams, T. E. E. Nutrient contents and efficiencies of beech and spruce saplings as influenced by competition and O-3/CO2 regime. European Journal of Forest Research. 128 (2), 117-128 (2009).
70. Rouhier, H. & Read, D. J. The role of mycorrhiza in determining the response of Plantago lanceolata to CO2 enrichment. New Phytologist. 139 (2), 367-373 (1998).
71. Seneweera, S. P. & Conroy, J. P. Growth, grain yield and quality of rice (Oryza sativa L.) in response to elevated CO2 and phosphorus nutrition (Reprinted from Plant nutrition for sustainable food production and environment, 1997). Soil Science and Plant Nutrition. 43 (SI), 1131-1136 (1997).
72. Shimono, H. *et al.* Lodging in rice can be alleviated by atmospheric CO_2 enrichment. *Agric. Ecosyst. Environ.* **118**, 223-230 (2007).
73. Singh SK, Badgujar GB, Reddy VR, Fleischer DH, Timlin DJ. Effect of phosphorus nutrition on growth and Physiology of cotton under ambient and elevated carbon dioxide. J Agronomy and Crop Science, 199, 436-448.
74. Syversten JP, Graham JH. Phosphorus supple and arbuscular mycorrhuizas increase growth and net gas exchange responses of two Citrus spp. Grown at elevated [CO2]. Plant Soil, 208, 209-219.
75. Temperton, V. M., Grayston S, Jackson G, Barton CVM, Millard P, Jarvis PG. Effects of elevated carbon dioxide concentration on growth and nitrogen fixation in *Alnus glutinosa* in a long-term field experiment. *Tree Physiol.* **23**, 1051-1059 (2003).
76. Teng, N. J., Wang J, Chen T, Wu X, Wang Y, Lin J. Elevated CO_2 induces physiological, biochemical and structural changes in leaves of *Arabidopsis thaliana*. *New Phytol.* **172**, 92-103 (2006).
77. Tissue, D. T. & Lewis, J. D. Photosynthetic responses of cottonwood seedlings grown in glacial through future atmospheric [CO2] vary with phosphorus supply. Tree Physiology. 30 (11), 1361-1372 (2010).
78. Tremblay, N., Yelle, S. & Gosselin, A. Effects of Co2 enrichment, nitrogen and phosphorus fertilization during transplant production growth and yield of celery. Canadian Journal of Plant Science. 68 (2), 571-571 (1988).
79. Utriainen, J., Janhunen, S., Helmisaari, H. S. & Holopainen, T. Biomass allocation, needle structural characteristics and nutrient composition in Scots pine seedlings exposed to elevated CO_2 and O_3 concentrations. *Trees* **14**, 475-484 (2000).
80. Walker, R. F., Johnson, D. W., Geisinger, D. R. & Ball, J. T. Growth, nutrition, and water relations of ponderosa pine in a field soil as influenced by long-term exposure to elevated atmospheric CO_2. *For. Ecol. Manage.* **137**, 1-11 (2000).
81. Watling JR, Press MC. How does the C_4 grass *Eragrostis pilosa* respond to elevated carbon dioxide and infection with the parasitic angiosperm *Striga hermonthica*? New Phytol. 1998;140:667–675.
82. Weigt, R. B., Raidl, S., Verma, R., Rodenkirchen, H., Gottlein, A. & Agerer, R. Effects of twice-ambient carbon dioxide and nitrogen amendment on biomass, nutrient contents and carbon costs of Norway spruce seedlings as influenced by mycorrhization with Piloderma croceum and Tomentellopsis submollis. Mycorrhiza. 21 (5), 375-391 (2011).
83. Wilsey, B. J., Mcnaughton, S. J. & Coleman, J. S. Will increases in atmospheric Co2 affect regrowth following grazing in c-4 grasses from tropical grasslands? a test with sporobolus-kentrophyllus. Oecologia. 99 (1-2), 141-144 (1994).
84. Winkler, J. B. & Herbst, M. Do plants of a semi-natural grassland community benefit from long-term CO_2 enrichment? *Basic Appl. Ecol.* **5**, 131-143 (2004).
85. Woodin, S., Graham, B., Killick, A., Skiba, U. & Cresser, M. Nutrient limitation of the long-term response of heather (Calluna vulgaris (L) Hull) to Co2 enrichment. New Phytologist. 122 (4), 635-642 (1992).

86. Wu, D. X., Wang, G. X., Bai, Y. F. & Liao, J. X. Effects of elevated CO_2 concentration on growth, water use, yield and grain quality of wheat under two soil water levels. *Agric. Ecosyst. Environ.* **104**, 493-507 (2004).
87. Xie ZB, Zhu JG, Zhang YL, Ma HL, Liu G, Han Y, Zeng Q, and Cai ZC. Responses of rice (*Oryza sativa*) growth and its C, N and P composition to FACE (Free-air Carbon Dioxide Enrichment) and N, P fertilization. *Chinese J. Appl. Ecol.* **13**, 1223-1230 (2002).
88. Xie, Z. B., Zhu, J. G., Pan, H. L., Ma, H. L., Liu, G., Georg, C. and Pang, J. 2004. Stimulated rice growth and decreased straw quality under free air CO2 enrichment (FACE). *In* Feng, C. G., Huang, P., Ma, Y., Wang, Y. J., Li, S. C. and Su, Q. (eds.) The Proceedings of the China Association for Science and Technology. Science Press, Beijing/New York. pp. 640–645.
89. Yamakawa, Y., Saigusa, M., Okada, M., Kobayashi, K. Nutrient uptake by rice and soil solution composition under atmospheric CO2 enrichment. Plant and Soil. 259 (1–2), 367–372 (2004)
90. Yan, X., Yu, D. & Li, Y. K. The effects of elevated CO_2 on clonal growth and nutrient content of submerge plant *Vallisneria spinulosa*. *Chemosphere* **62**, 595-601 (2006).
91. Yang, L. X., Huang J, Yang HJ, Dong GC, Liu HJ, Liu G, Zhu JG, Wang Y. Seasonal changes in the effects of free-air CO_2 enrichment (FACE) ion nitrogen (N) uptake and utilization of rice at three levels of N fertilization. *Field Crops Res.* **100**, 189-199 (2007).
92. Yang, L. X. Wang Y, Dong G, Gu H, Huang J, Zhu J, Yang H, Liu G, Han Y. The impact of free-air CO_2 enrichment (FACE) and nitrogen supply on grain quality of rice. *Field Crops Res.* **102**, 128-140 (2007).
93. Zhang, S., Dang, Q. Effects of carbon dioxide concentration and nutrition on photosynthetic functions of white birch seedlings. Tree Physiology 26 (11), 1457–1467 (2006).
