# Supplementary material for: Increasing atmospheric CO2 concentrations correlate with declining nutritional status of European forests
Source: Commun Biol. 2020 Mar 13;3:125. doi: 10.1038/s42003-020-0839-y (PMC7070084; doi:10.1038/s42003-020-0839-y)
Supplement: Supplementary file 2 — Reporting Summary [file 42003_2020_839_MOESM2_ESM.pdf]

nature research

Corresponding author(s): Josep Penuelas

Last updated by author(s): Feb 3, 2020

Reporting Summary

Nature Research wishes to improve the reproducibility of the work that we publish. This form provides structure for consistency and transparency in reporting. For further information on Nature Research policies, see Authors & Referees and the Editorial Policy Checklist.

Statistics

For all statistical analyses, confirm that the following items are present in the figure legend, table legend, main text, or Methods section.

n/a	Confirmed	
☐	☒	The exact sample size (*n*) for each experimental group/condition, given as a discrete number and unit of measurement
☐	☒	A statement on whether measurements were taken from distinct samples or whether the same sample was measured repeatedly
☐	☒	The statistical test(s) used AND whether they are one- or two-sided<br>*Only common tests should be described solely by name; describe more complex techniques in the Methods section.*
☐	☒	A description of all covariates tested
☐	☒	A description of any assumptions or corrections, such as tests of normality and adjustment for multiple comparisons
☐	☒	A full description of the statistical parameters including central tendency (e.g. means) or other basic estimates (e.g. regression coefficient) AND variation (e.g. standard deviation) or associated estimates of uncertainty (e.g. confidence intervals)
☐	☒	For null hypothesis testing, the test statistic (e.g. *F*, *t*, *r*) with confidence intervals, effect sizes, degrees of freedom and *P* value noted<br>*Give P values as exact values whenever suitable.*
☒	☐	For Bayesian analysis, information on the choice of priors and Markov chain Monte Carlo settings
☒	☐	For hierarchical and complex designs, identification of the appropriate level for tests and full reporting of outcomes
☐	☒	Estimates of effect sizes (e.g. Cohen's *d*, Pearson's *r*), indicating how they were calculated

Our web collection on statistics for biologists contains articles on many of the points above.

Software and code

Policy information about availability of computer code

Data collection	R
Data analysis	R

For manuscripts utilizing custom algorithms or software that are central to the research but not yet described in published literature, software must be made available to editors/reviewers. We strongly encourage code deposition in a community repository (e.g. GitHub). See the Nature Research guidelines for submitting code & software for further information.

Data

Policy information about availability of data

All manuscripts must include a data availability statement. This statement should provide the following information, where applicable:

- Accession codes, unique identifiers, or web links for publicly available datasets
- A list of figures that have associated raw data
- A description of any restrictions on data availability

All data and codes are available upon request to the corresponding author, Professor Josep Penuelas.

Field-specific reporting

Please select the one below that is the best fit for your research. If you are not sure, read the appropriate sections before making your selection.

☐ Life sciences ☐ Behavioural & social sciences ☒ Ecological, evolutionary & environmental sciences

For a reference copy of the document with all sections, see nature.com/documents/nr-reporting-summary-flat.pdf

Ecological, evolutionary & environmental sciences study design

All studies must disclose on these points even when the disclosure is negative.

Study description

Here we show forest foliar concentrations of N, P, K, S and Mg decreased significantly in Europe by 5, 11, 8, 6 and 7%, respectively during the last three decades. The decrease in nutritional status was especially large in Mediterranean and temperate forests.

Research sample

Seven dominant species, Pinus sylvestris, Picea abies, Abies alba, Fagus sylvatica, Quercus robur, Quercus petraea and Quercus ilex, for the entire Europe and for northern, central and southern Europe.

Sampling strategy

We used foliar and growth data of the International Co-operative Programme on Assessment and Monitoring of Air Pollution Effects on Forests operating under the UNECE Convention on Long-range Transboundary Air Pollution (CLRTAP) (ICP Forests). Activities under ICP Forests are conducted in 42 states using harmonized sampling and analysis following the manuals on Sampling and Analysis of needles and leaves – Part XII (http://www.icp-forests.org/pdf/manual/2010/Manual_2010_Foliage.pdf) and Tree growth – Part V (http://www.icp-forests.org/pdf/manual/2010/Manual_2010_Growth.pdf). In this study, we have gathered all the available data of foliar N, P, K, S, Ca and Mg concentrations in annual series. We have used data from 28 countries with a total of 528 different plots and with 506 of these plots with the canopy clearly dominated by one tree species, 21 co-dominated by two tree species, and 1 plot co-dominated by three tree species.

Foliar samples were taken at least biannually at the intensive forest monitoring plots (Level II) from the living crown of the dominant canopy layer providing information on the nutrient status of one or more species per plot. The analysis was done for 1000 needles or 100 leaves covering a range of elements (for detailed information please refer to the ICP Forest Manual). Briefly, in each plot a minimum of 5 dominant trees were randomly selected avoiding the trees used in crown assessment, so to avoid crown damage of these trees. A composite sample by species was made by mixing equal quantities of each sample per plot after drying. Sampled leaves were mature current year leaves or needles. Only in the case of evergreens such as Q. ilex both mature (non-senescent) one- and second-year leaves were sampled. The sample analyses procedure was based on homologated methods, basically direct C and N determination by elemental-analyser (Kjeldhal method was also allowed for N determination), whereas for the other elements the most common procedure was based on acid digestion with nitric acid or nitric acid mixtures (43-46%) followed by wet ashing (40%) (De Vries et al. 2010) and posterior determination, mostly by inductively coupled plasma coupled to atomic emission spectrometry (ICP-AES). Quality assurance and assessment of the analytical process was controlled by the regular organization of Inter-laboratory Comparisons by the Forest Foliar Co-ordinating Centre. The laboratory results were considered of sufficient quality when the laboratory receives a qualification for the concerning parameter(s) after participation in the Inter-laboratory comparisons. The growth survey assessed the diameter at breast height (dbh) for the dominant trees at a five or ten yearly interval. Additionally, permanent girth bands provided annual data at dbh.

For atmospheric CO_2 concentration, we used monthly data from the Mauna Loa Observatory, available online (http://scrippsco2.ucsd.edu/data/atmospheric_co2/) and provided by the Scripps Institution of Oceanography (Scripps CO_2 programme). We calculated the annual averages to use in our statistical analyses. We obtained the meteorological-climate data from CRU TS v3.25 of the Climatic Research Unit (Harris et al., 2014). Annual data for N (NO_3^- + NH_4^+) and S (SO_4^-) atmospheric deposition were extracted from the European Monitoring and Evaluation Programme (EMEP) with a spatial resolution of 0.1 × 0.1° for longitude and latitude, and the MSC-W chemical transport model developed to estimate regional atmospheric dispersion and deposition of acidifying and eutrophying compounds of N and S over Europe.

Data collection

We used foliar and growth data of the International Co-operative Programme on Assessment and Monitoring of Air Pollution Effects on Forests operating under the UNECE Convention on Long-range Transboundary Air Pollution (CLRTAP) (ICP Forests). Activities under ICP Forests are conducted in 42 states using harmonized sampling and analysis following the manuals on Sampling and Analysis of needles and leaves – Part XII (http://www.icp-forests.org/pdf/manual/2010/Manual_2010_Foliage.pdf) and Tree growth – Part V (http://www.icp-forests.org/pdf/manual/2010/Manual_2010_Growth.pdf). In this study, we have gathered all the available data of foliar N, P, K, S, Ca and Mg concentrations in annual series. We have used data from 28 countries with a total of 528 different plots and with 506 of these plots with the canopy clearly dominated by one tree species, 21 co-dominated by two tree species, and 1 plot co-dominated by three tree species.

Foliar samples were taken at least biannually at the intensive forest monitoring plots (Level II) from the living crown of the dominant canopy layer providing information on the nutrient status of one or more species per plot. The analysis was done for 1000 needles or 100 leaves covering a range of elements (for detailed information please refer to the ICP Forest Manual). Briefly, in each plot a minimum of 5 dominant trees were randomly selected avoiding the trees used in crown assessment, so to avoid crown damage of these trees. A composite sample by species was made by mixing equal quantities of each sample per plot after drying. Sampled leaves were mature current year leaves or needles. Only in the case of evergreens such as Q. ilex both mature (non-senescent) one- and second-year leaves were sampled. The sample analyses procedure was based on homologated methods, basically direct C and N determination by elemental-analyser (Kjeldhal method was also allowed for N determination), whereas for the other elements the most common procedure was based on acid digestion with nitric acid or nitric acid mixtures (43-46%) followed by wet ashing (40%) (De Vries et al. 2010) and posterior determination, mostly by inductively coupled plasma coupled to atomic emission spectrometry (ICP-AES). Quality assurance and assessment of the analytical process was controlled by the regular organization of Inter-laboratory Comparisons by the Forest Foliar Co-ordinating Centre. The laboratory results were considered of sufficient quality when the laboratory receives a qualification for the concerning parameter(s) after participation in the Inter-laboratory comparisons. The growth survey assessed the diameter at breast height (dbh) for the dominant trees at a five or ten yearly interval. Additionally, permanent girth bands provided annual data at dbh.

For atmospheric CO_2 concentration, we used monthly data from the Mauna Loa Observatory, available online (http://scrippsco2.ucsd.edu/data/atmospheric_co2/) and provided by the Scripps Institution of Oceanography (Scripps CO_2 programme). We calculated the annual averages to use in our statistical analyses. We obtained the meteorological-climate data from CRU TS v3.25 of the Climatic Research Unit (Harris et al., 2014). Annual data for N (NO_3^- + NH_4^+) and S (SO_4^-) atmospheric deposition were extracted from the European Monitoring and Evaluation Programme (EMEP) with a spatial resolution of 0.1 × 0.1° for longitude and latitude, and the MSC-W chemical transport model developed to estimate regional atmospheric dispersion and deposition of acidifying and eutrophying compounds of N and S over Europe.

We used foliar and growth data of the International Co-operative Programme on Assessment and Monitoring of Air Pollution Effects on Forests operating under the UNECE Convention on Long-range Transboundary Air Pollution (CLRTAP) (ICP Forests). Activities under ICP Forests are conducted in 42 states using harmonized sampling and analysis following the manuals on Sampling and Analysis of needles and leaves – Part XII (http://www.icp-forests.org/pdf/manual/2010/Manual_2010_Foliage.pdf) and Tree growth – Part V (http://www.icp-forests.org/pdf/manual/2010/Manual_2010_Growth.pdf). In this study, we have gathered all the available data of foliar N, P, K, S, Ca and Mg concentrations in annual series. We have used data from 28 countries with a total of 528 different plots and with 506 of these plots with the canopy clearly dominated by one tree species, 21 co-dominated by two tree species, and 1 plot co-dominated by three tree species.
Foliar samples were taken at least biannually at the intensive forest monitoring plots (Level II) from the living crown of the dominant canopy layer providing information on the nutrient status of one or more species per plot. The analysis was done for 1000 needles or 100 leaves covering a range of elements (for detailed information please refer to the ICP Forest Manual). Briefly, in each plot a minimum of 5 dominant trees were randomly selected avoiding the trees used in crown assessment, so to avoid crown damage of these trees. A composite sample by species was made by mixing equal quantities of each sample per plot after drying. Sampled leaves were mature current year leaves or needles. Only in the case of evergreens such as Q. ilex both mature (non-senescent) one- and second-year leaves were sampled. The sample analyses procedure was based on homologated methods, basically direct C and N determination by elemental-analyser (Kjeldhal method was also allowed for N determination), whereas for the other elements the most common procedure was based on acid digestion with nitric acid or nitric acid mixtures (43-46%) followed by wet ashing (40%) (De Vries et al. 2010) and posterior determination, mostly by inductively coupled plasma coupled to atomic emission spectrometry (ICP-AES). Quality assurance and assessment of the analytical process was controlled by the regular organization of Inter-laboratory Comparisons by the Forest Foliar Co-ordinating Centre. The laboratory results were considered of sufficient quality when the laboratory receives a qualification for the concerning parameter(s) after participation in the Inter-laboratory comparisons. The growth survey assessed the diameter at breast height (dbh) for the dominant trees at a five or ten yearly interval. Additionally, permanent girth bands provided annual data at dbh.
For atmospheric CO2 concentration, we used monthly data from the Mauna Loa Observatory, available online (http://scrippsco2.ucsd.edu/data/atmospheric_co2/) and provided by the Scripps Institution of Oceanography (Scripps CO2 programme). We calculated the annual averages to use in our statistical analyses. We obtained the meteorological-climate data from CRU TS v3.25 of the Climatic Research Unit (Harris et al., 2014). Annual data for N (NO3− + NH4+) and S (SO4−) atmospheric deposition were extracted from the European Monitoring and Evaluation Programme (EMEP) with a spatial resolution of 0.1 × 0.1° for longitude and latitude, and the MSC-W chemical transport model developed to estimate regional atmospheric dispersion and deposition of acidifying and eutrophying compounds of N and S over Europe.

Timing and spatial scale

Last three decades (1990-2016)
Europe

Data exclusions

No data excluded

Reproducibility

All attempts to repeat the analyses were successful. For example, the neural networks had two hidden layers with 128 units each with rectified linear activation functions. We repeated the process 1000 times making predicted maps and averaging the results.

Randomization

Not relevant to our study. See Methods please.

Blinding

Not relevant to our study. See Methods please.

Did the study involve field work? ☐ Yes ☒ No

Reporting for specific materials, systems and methods

We require information from authors about some types of materials, experimental systems and methods used in many studies. Here, indicate whether each material, system or method listed is relevant to your study. If you are not sure if a list item applies to your research, read the appropriate section before selecting a response.

Materials & experimental systems

n/a	Involved in the study
☒	☐ Antibodies
☒	☐ Eukaryotic cell lines
☒	☐ Palaeontology
☒	☐ Animals and other organisms
☒	☐ Human research participants
☒	☐ Clinical data

Methods

n/a	Involved in the study
☒	☐ ChIP-seq
☒	☐ Flow cytometry
☒	☐ MRI-based neuroimaging
